# Maternal plasma folate impacts differential DNA methylation in an epigenome-wide meta-analysis of newborns

**DOI:** 10.1038/ncomms10577

**Published:** 2016-02-10

**Authors:** Bonnie R. Joubert, Herman T. den Dekker, Janine F. Felix, Jon Bohlin, Symen Ligthart, Emma Beckett, Henning Tiemeier, Joyce B. van Meurs, Andre G. Uitterlinden, Albert Hofman, Siri E. Håberg, Sarah E. Reese, Marjolein J. Peters, Bettina Kulle Andreassen, Eric A. P. Steegers, Roy M. Nilsen, Stein E. Vollset, Øivind Midttun, Per M. Ueland, Oscar H. Franco, Abbas Dehghan, Johan C. de Jongste, Michael C. Wu, Tianyuan Wang, Shyamal D. Peddada, Vincent W. V. Jaddoe, Wenche Nystad, Liesbeth Duijts, Stephanie J. London

**Affiliations:** 1 Division of Intramural Research, National Institute of Environmental Health Sciences, National Institutes of Health, Department of Health and Human Services, Research Triangle Park, North Carolina 27709, USA; 2 The Generation R Study Group, Erasmus MC, University Medical Center Rotterdam, Rotterdam 3000 CA, Netherlands; 3 Department of Pediatrics, Division of Respiratory Medicine, Erasmus MC, University Medical Center Rotterdam, Rotterdam 3000 CA, Netherlands; 4 Department of Epidemiology, Erasmus MC, University Medical Center Rotterdam, Rotterdam 3000 CA, Netherlands; 5 Department of Pediatrics, Erasmus MC, University Medical Center Rotterdam, Rotterdam 3000 CA, Netherlands; 6 Norwegian Institute of Public Health, Oslo 0403, Norway; 7 Department of Applied Sciences, School of Environmental and Life Sciences, University of Newcastle, Ourimbah, New South Wales 2258, Australia; 8 Food and Nutrition Flagship, CSIRO, North Ryde, New South Wales 2113, Australia; 9 Department of Psychiatry, Erasmus MC, University Medical Center Rotterdam, Rotterdam 3000 CA, Netherlands; 10 Department of Internal Medicine, Erasmus MC, University Medical Center Rotterdam, Rotterdam 3000 CA, Netherlands; 11 Department of Clinical Molecular Biology, Institute of Clinical Medicine, University of Oslo, Oslo 0316, Norway; 12 Department of Obstetrics and Gynaecology, Erasmus MC, University Medical Center Rotterdam, Rotterdam 3000 CA, Netherlands; 13 Department of Research and Development, Centre for Clinical Research, Haukeland University Hospital, Bergen 5021, Norway; 14 Department of Global Public Health and Primary Care, University of Bergen, Bergen 5018, Norway; 15 Bevital A/S, Laboratoriebygget, Bergen 5018, Norway; 16 Department of Clinical Science, University of Bergen, Bergen 5018, Norway; 17 Laboratory of Clinical Biochemistry, Haukeland University Hospital, Bergen 5018, Norway; 18 Public Health Sciences Program, Fred Hutchinson Cancer Research Center, Seattle, Washington 98109, USA

## Abstract

Folate is vital for fetal development. Periconceptional folic acid supplementation and food fortification are recommended to prevent neural tube defects. Mechanisms whereby periconceptional folate influences normal development and disease are poorly understood: epigenetics may be involved. We examine the association between maternal plasma folate during pregnancy and epigenome-wide DNA methylation using Illumina's HumanMethyl450 Beadchip in 1,988 newborns from two European cohorts. Here we report the combined covariate-adjusted results using meta-analysis and employ pathway and gene expression analyses. Four-hundred forty-three CpGs (320 genes) are significantly associated with maternal plasma folate levels during pregnancy (false discovery rate 5%); 48 are significant after Bonferroni correction. Most genes are not known for folate biology, including *APC2*, *GRM8*, *SLC16A12*, *OPCML*, *PRPH*, *LHX1*, *KLK4* and *PRSS21.* Some relate to birth defects other than neural tube defects, neurological functions or varied aspects of embryonic development. These findings may inform how maternal folate impacts the developing epigenome and health outcomes in offspring.

## Abstract (web summary)

Folic acid is routinely recommended for women trying to conceive to ensure proper fetal development. Here, the authors perform a large epigenomics study to examine which fetal epigenetic changes are associated with varied maternal plasma folate levels.

## Graphical abstract

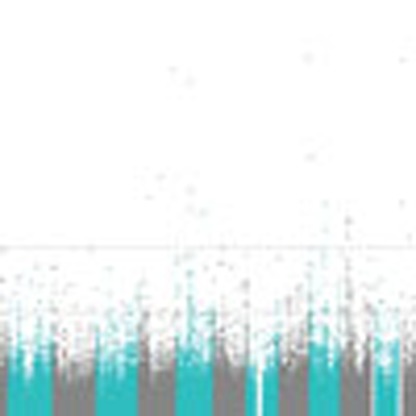

Folate (vitamin B9) is vital for fetal development. Folic acid supplementation at 0.4 mg per day or higher is recommended worldwide before and in the very early stages of pregnancy to reduce the incidence of neural tube defects (NTDs). Over 50 countries have introduced programs to fortify the food supply with folic acid to increase folate levels in women of childbearing age^1^. Rates of NTDs have clearly decreased following fortification^2^ and there is increasing interest in the possibility that higher maternal folate prevents additional birth defects including oral clefts, cardiac defects and others^3^. A large international trial has been launched of supplementation with 4 mg versus the standard 0.4 mg to attempt to address these questions^3^.

Other beneficial effects of higher maternal folate levels have been reported in humans. These include reduced risk of low birth weight, pre-term delivery, language delay, leukaemia, childhood brain tumours and autism, although the evidence is inconsistent^4^,^5^. In the United States, food fortification has led to an increase in folate intake twice as large as anticipated^6^, and therefore concern has been raised about possible adverse effects, such as cancer in adults, as a result of this population-wide intervention^1^. Furthermore, higher folic acid intake during pregnancy has been associated with an increased risk of childhood retinoblastoma and early respiratory illness^4^.

The mechanisms whereby folic acid prevents NTDs and potentially other birth defects and later health outcomes are poorly understood^7^ but could involve epigenetic changes. Folate is a critical component in the one-carbon metabolism pathway providing methyl groups for a range of biochemical reactions, including methylation of DNA^8^. DNA methylation is an important epigenetic determinant of gene expression, and differential methylation has been associated with multiple diseases^9^. Periconceptional maternal folate levels may alter methylation patterns established *in utero* that are vital for fetal development, which could impact later health outcomes in the offspring.

In mouse models, *in utero* dietary methyl donor supplementation has been associated with altered methylation patterns and disease phenotypes^4^. The brains of human fetuses with NTDs had lower global methylation compared with controls, which was positively correlated with maternal folate levels^10^. With respect to gene-specific differential methylation, perinatal folate has also been associated with differential methylation in specific imprinted genes, such as *IGF2* and *H19*, in offspring, but reported results are inconsistent^11^. The only published study using a platform with reasonable genome-wide coverage, the Illlumina HumanMethyl450 Beadchip (450 K), investigated 23 subjects and reported that folic acid supplementation during pregnancy was related to differential methylation upstream of the gene *ZFP57*, which plays a central role in the regulation and maintenance of imprinting^12^.

Some countries, such as Norway and the Netherlands, do not fortify the food supply with folic acid. These populations may be particularly useful for examining the biological implications of periconceptional folic acid supplementation on offspring health, as greater variability in the dose and the source of folate may exist compared with fortified populations.

To better understand the biological implications of folate status on the developing fetus, we examine the association between maternal plasma folate during pregnancy and epigenome-wide differential DNA methylation in newborn cord blood using the Illumina HumanMethyl450 (450 K) Beadchip. We include 1,988 newborns from two European pregnancy cohorts of Caucasian ancestry, the Norwegian Mother and Child Cohort Study (MoBa), and the Generation R Study (Generation R). We combine results using meta-analysis. Secondary pathway analyses and gene expression analyses are also explored.

## Results

### Study characteristics

In MoBa participants (*N=*1,275), maternal plasma folate levels ranged from 1.6 to 53.2 nmol l^−1^ (mean*=*11.9). The maternal plasma folate levels in Generation R (*N=*713) ranged from 4.1 to 45.3 nmol l^−1^ (mean*=*20.3; Table 1). The mean maternal age was ∼30 years for both cohorts. Approximately, 15% of MoBa mothers and 25% of Generation R mothers smoked during the pregnancy and over 60% obtained college or more advanced levels of education in both studies (Table 1).

### Meta-analysis

**Figure 1:**
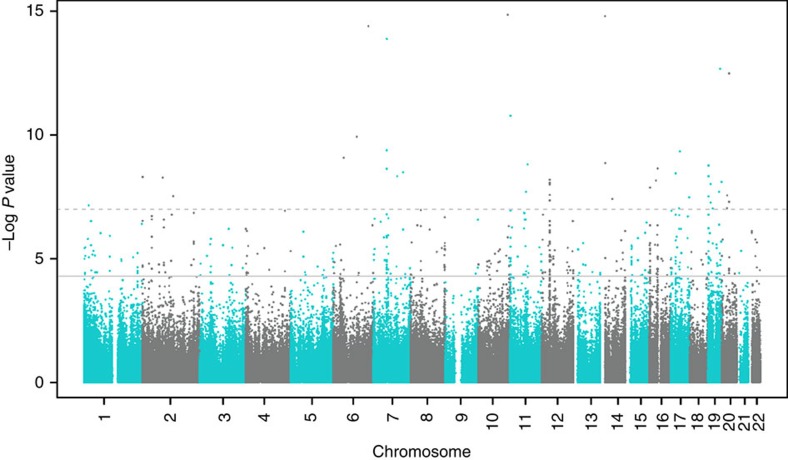
Association between maternal plasma folate and DNA methylation in newborn cord blood: meta-analysis results for MoBa (*N*=1,275) and Generation R (*N*=713) cohorts. The uncorrected –log10(*P* values) are plotted by CpG genomic position. Multiple testing was accounted for using the false discovery rate (FDR) procedure by Benjamini and Hochberg. A total of 443 CpGs were considered FDR significant (solid horizontal line); 48 CpGs were also Bonferroni significant (dashed horizontal line).

Our meta-analysis of the association between maternal plasma folate levels during pregnancy and differential DNA methylation in newborn cord blood, adjusted for covariates, resulted in 443 false discovery rate (FDR)-significant CpGs (Benjamini and Hochberg FDR-corrected *P* (*P*_BH_)<0.05; Fig. 1). Genes with two or more FDR-significant CpGs, where at least one CpG was within the gene, were prioritized for further discussion (Table 2). Results for all FDR-significant CpGs are shown in Supplementary Data 1 (sorted by the uncorrected *P* value) and Supplementary Data 2 (sorted by chromosome and position). The vast majority of the FDR-significant CpGs were robust to covariate adjustment as well as adjustment for cell type; coefficients from the unadjusted, covariate-adjusted, and covariate- and cell-type-adjusted models were in the same direction and had a similar magnitude of effect (Supplementary Data 1 and 2). More detailed gene information is provided in Supplementary Table 1. The genomic inflation factor (lambda)^13^ values for the unadjusted, covariate-adjusted, and covariate- and cell-type-adjusted models were 0.96, 1.07 and 1.16, respectively (Supplementary Figs 1–3). Among the 443 FDR-significant CpGs in the covariate-adjusted meta-analysis model, increasing levels of maternal plasma folate during pregnancy were associated with decreased methylation of 416 (94%) and increased methylation of 27 (6%) CpGs. There were 48 CpGs that also met the strict Bonferroni threshold for statistical significance (*P*<1.19 × 10^−7^, correcting for 419,905 tests). The direction of effects for the statistically significant CpGs was largely consistent in the MoBa and Generation R populations (Table 2; Supplementary Data 1 and 2).

### Sensitivity analyses

We considered whether vitamin B12, a co-factor with folate in one-carbon metabolism, contained in most multivitamins, along with other B vitamins such as B6 and riboflavin, might confound associations between folate and methylation. Vitamin B12 and folate levels were modestly positively correlated (Spearman correlation 0.11 in MoBa, 0.14 in Generation R, *P*<0.001 for both). When we adjusted for vitamin B12, the coefficients for folate in relation to methylation changed only minimally (median change 4.9%, 25–75th percentile 2.3–8.2%, *N=*1,933 subjects). In addition to the consistency of effect estimates after adjustment, results remained statistically significant for 376 (85%) at Bonferroni correction for 443 tests, *P*<1.13 × 10^−4^, and all 443 CpGs had *P*<9 × 10^−4^ (Supplementary Data 3). Thus, vitamin B12 does not confound the folate–methylation associations we observed.

Women with higher folate levels, which largely reflect supplement use, might be more likely to take multivitamins and/or separate supplements such as cod liver or fish oils that are common in Norway. However, vitamin D (total of D2 and D3) levels were modestly correlated with folate levels (Spearman correlation coefficient=0.14 in MoBa, 0.23 in Generation R, *P*<0.001 for both cohorts). Adjustment for vitamin D only minimally altered effect estimates for folate in relation to methylation (median absolute value of change 7.3%, 25–75th percentile 3.3–12.3%). Despite the reduction in power due to the smaller sample size for these adjusted analyses (*N=*1,664), 70% of CpGs significantly related to folate in the main model remained Bonferroni significant after adjustment for vitamin D (308 with *P*<1.13 × 10^−4^; Supplementary Data 3).

We performed additional analyses adjusting for two single-nucleotide polymorphisms (SNPs) in the *MTHFR* gene that influence one-carbon metabolism and are correlated with plasma folate: rs1801133 and rs1801131 (refs 14, 15). These SNPs are in moderate linkage disequilibrium with each other (*r*^2^=0.20–21 in the two studies). Adjustment for these two SNPs made little difference in the effect estimates compared with the main model; median change in coefficient=3.8% (25–75th percentile=2.0–6.9%) and 85% of CpGs remained statistically significant despite reduction in sample size to 1,880 (*P*<1.13 × 10^−4^, correction for 443 tests). Thus, these genetic variants do not confound the relationship between folate and methylation.

Homocysteine, unlike folate or vitamin B12, is not a nutrient that plays a role as a methyl donor or carrier, but is a product formed during transmethylation in the one-carbon metabolism cycle. It could be regarded as an intermediate on the causal pathway between folate and methylation. In addition, like plasma folate, it is an excellent marker of folate status. Homocysteine was strongly correlated with maternal plasma folate in MoBa (Spearman correlation=−0.49, *P*<0.001) and moderately correlated in Generation R (Spearman correlation=−0.24, *P*<0.001), making it challenging to estimate independent effects. Given these various factors, inclusion of homocysteine in the model led to a moderate change in the coefficients for folate in relation to methylation (median change 10.7%, 25–75th percentile 5.8–17.2%. *N=*1,931 subjects) and only 137 (31%) CpGs remained statistically significant (*P*<1.13 × 10^−4^, correction for 443 tests).

We also examined whether the associations with methylation seen for maternal folate levels are also seen for newborn folate levels in a subset of 572 subjects in Generation R. Thus, this analysis is not well powered compared with our maternal folate analysis with 1,988 subjects. However, of the 443 FDR-significant findings for maternal folate in the meta-analysis there were 60 (14%) with nominal *P* values<0.05 for newborn folate which is higher than the 5% expected by chance alone (Kolmogorov *P*<1.2 × 10^−13^). This supports the interpretation that some similar loci are differentially methylated in response to infant folate, although we were severely underpowered to address this properly.

### Pathway analysis

Pathway analysis with the FDR-significant CpGs showed strong and consistent enrichment of fundamental development pathways and of neurodevelopmental pathways (Supplementary Tables 2–4). The biological processes implicated from the DAVID pathway analysis included cell development, embryonic morphogenesis, development, regulation of multicellular organismal processes, cell–cell signalling, embryonic development, forebrain development and, notably, neural tube development (Supplementary Table 2). Ingenuity Pathway Analysis (IPA) results indicated pathways related to nervous system development and function, cell–cell signalling and basic developmental processes (Supplementary Table 3). Gene ontology enrichment analysis and visualization tool results included pathways related to the synaptic signalling, cell–cell signalling, regulation of cAMP biosynthetic process, single-organism behaviour, single-organism signalling, signalling, regulation of gastrulation and the regulation of nervous system development (Supplementary Table 4).

### Methylation expression analysis

Of the 365 CpGs associated with folate that we were able to match to a gene transcript (±250 kb), 43 CpGs were significantly associated with altered expression of nearby genes (*P*_BH_<0.1). For most CpGs, increased methylation was associated with decreased gene expression (Supplementary Table 5).

## Discussion

Our study is the largest to date using the Illumina 450 K epigenome-wide platform to evaluate the impact of maternal plasma folate levels during pregnancy on DNA methylation in newborns. We meta-analysed results from two population-based birth cohort studies in Northern Europeans that measured DNA methylation using the same platform. We observed epigenome-wide FDR-significant associations between maternal plasma folate and DNA methylation in cord blood at 443 CpGs.

It is notable that many of the implicated genes have functional relevance to various developmental pathways. Some are relevant to NTDs, the indication for maternal folic acid supplementation, and others to distinct developmental conditions that have not been previously associated with maternal folate levels. Additional genes we identified have been implicated in conditions where there is some concern about possible adverse effects of higher folate levels, such as breast cancer progression^16^. Due to the large number of genes significantly differentially methylated in relation to folate (Supplementary Data 1 and 2), we focus this discussion primarily on genes with two or more CpGs at genome-wide significance after FDR correction (*P*_BH_<0.05) where at least one CpG is within the gene (Table 2).

We observed the largest number (nine) of statistically significant CpGs mapping to the gene adenomatosis polyposis coli 2 gene (*APC2*). *APC2* is expressed in both human fetal and adult brain^17^ and in the peripheral nervous system^18^. It plays a critical role in the brain development in several model systems^19^. *APC2* may also play a role in cancer aetiology. A homologue of the tumour suppressor gene *APC*^20^, *APC2*, is involved in the regulation of the Wnt signalling pathway, which impacts both normal development and tumorigenesis^21^. Studies in mice have reported associations between periconceptional maternal folate and methylation of *APC* genes^22^. In two human breast cancer lines, folate leads to methylation-mediated silencing of *APC* and other tumour suppressor genes, raising concern about the risk of tumour progression^23^. Thus, folate-related methylation of *APC2* during fetal development could impact both pathways of neurodevelopment and carcinogenesis.

*GRM8* encodes a glutamate receptor that interacts with L-glutamate, the major excitatory neurotransmitter in the central nervous system. Glutamatergic neurotransmission is ubiquitous in normal brain function^24^ and is perturbed in various neuropathologies. In humans, copy-number variations of *GRM8* have been associated with neurodevelopmental disorders such as attention-deficit hyperactivity disorder^25^ and autism spectrum disorder^26^.

A number of genes we identified as differentially methylated in newborns in relation to maternal folate are known to harbour mutations that have been causally implicated in various developmental abnormalities other than NTDs, the indication for folic acid supplementation in pregnancy. These include several with two or more statistically significant CpGs (Table 2) such as *SLC16A12*, implicated in juvenile cataracts with microcornea and renal glucosuria^27^; and *KLK4*, implicated in the dental malformation amelogenesis imperfecta^28^. Mutations in *LHX1* have been associated with abnormalities in uterine development^29^, and recent evidence suggests an important role in retinal development^30^. Several genes with one CpG at genome-wide statistical significance (Supplementary Data 1 and 2) also harbour mutations that are causal for various development malformations. These include *IHH* involved in skeletal malformations, *ROBO3* involved in horizontal gaze palsy with progressive scoliosis, *PCSK9* involved in familial hypercholesterolemia, *FAM83H* related to amelogenesis imperfecta type 3 and *GJA3* associated with congenital cataracts. Taken together, these findings suggest a role for periconceptional folate levels in birth defects not previously known to be related to this nutrient.

Our agnostic evaluation of maternal folate levels and DNA methylation in newborns also identified genes related to various neurologic diseases. Genetic variation in *OPCML* and *PRPH* has been associated with the neurodegenerative disease amyotrophic lateral sclerosis^31^,^32^. In genome-wide association studies, *CSMD1* has been associated with schizophrenia and autism^33^.

Some previous studies of folate and methylation have examined the *H19* imprinted region^11^,^34^. We identified three significant CpGs located 45–48-Kb upstream of *H19* among 77 CpGs on the platform that are within 48 up- or downstream of *H19*.

The largest number of statistically significant associations at any locus, 31, are on chromosome 12 and, based on our extended annotation, are nearest to *ALG10*. Two CpGs are 262–573-kb upstream; the other 29 CpGs are 261–573-kb downstream. None are in *ALG10*. Most are in a CpG island near the centromere and there are no features that suggest functional impact.

In the only previous study using the 450 K platform, Amarasekera *et al*^12^ reported differential methylation in relation to maternal folate in a 923-bp region on chromosome 6, 3-kb upstream of *ZFP57.* Our studies differ in sample sizes, design and analysis methods. However, when we evaluate the 20 CpGs that map to *ZFP57*, we find 5 with uncorrected *P* values of 0.05 or smaller—more than would be expected by chance alone. Thus, our data provide support for association at this locus.

From correlation analysis of 450 K methylation data and gene expression in white blood cells in adults, after correction for multiple testing, 43 CpGs that we implicated in relation to maternal folate were also related to expression of nearby genes (Supplementary Table 5). Although correlation of 450 K methylation with gene expression in the same newborn samples would have been preferable, we were only able to examine correlations in a population of Dutch adults. The most statistically significant correlation between methylation and gene expression was observed for the gene *PRSS21* (protease serine 21 (testisin)); four CpGs were both significantly associated with maternal folate (Table 2) and expression of this gene (Supplementary Table 5). *PRSS21* is a tumour suppressor gene silenced by aberrant methylation in testicular germ cell tumours^35^. Testicular germ cell tumours are diagnosed in early adulthood and can manifest as early as 15 years of age. Prenatal origin of this tumour has been proposed^36^; perhaps, methylation *in utero*, influenced by maternal folate levels, could play a role in this pathogenesis.

Because other important factors in one-carbon metabolism could potentially explain associations between folate levels and DNA methylation in cord blood, we performed various sensitivity analyses (Supplementary Data 3). On the basis of these analyses, vitamin B12 does not confound the folate–methylation association. This lack of confounding by B12 should extend to other B vitamins such as B6 and riboflavin that are present in multivitamins along with B12. We did not have data in both studies on choline, a nutrient that can serve as a source of one-carbon units. However, in MoBa, where choline was measured, there was no correlation with folate levels (Spearman correlation=−0.034, *P*=0.23) and thus choline should not confound associations between folate and methylation. Vitamin D is not part of the one-carbon metabolism cycle but might impact methylation by other mechanisms^37^. We performed analyses in a subsample taking vitamin D into account as proxy for intake of other supplements or possibly healthy dietary patterns and observed no major differences in results. Adjustment of the folate–methylation association for homocysteine, a product formed in one-carbon metabolism that is itself an excellent marker of folate status, resulted in a substantial reduction in the number of statistically significant findings. Although caution is required, both because folate and homocysteine are correlated, and because they operate together in a cycle rather than a clear unidirectional pathway, this attenuation could be interpreted as homocysteine, at least in part, mediating some of the associations between folate and methylation.

Given the role of folate as a major provider of methyl groups in the one-carbon metabolism pathway, our finding of reduced methylation with higher folate at the majority of the implicated CpGs may seem counterintuitive. However, methyl groups from the one-carbon metabolism pathway are used in a range of biological processes and the complex interactions of these systems may not necessarily result in linear relationships. Indeed, there is evidence that effects of folate on folate-dependent enzymes may switch directions at the higher intracellular concentrations that may accompany folic acid supplementation^38^. Folic acid, in vitamin supplements or food fortification, is a synthetic folate with possible effects that differ from those of natural occurring folate species. There is recent evidence that folic acid interferes with the inhibitory effect of *S*-adenosylmethionine (SAM) on methylenetetrahydrofolate reductase (MTHFR)^39^ and may inhibit MTHFR activity, thereby reducing the amount of 5-methyl-tetrahydrofolate, SAM and the SAM/*S*-adenosylhomocysteine (SAH) ratio^40^. The SAM/SAH ratio has been referred to as the methylation potential; low SAM/SAH ratio may decrease DNA methylation. This may explain the inverse relationship we observe in our study but additional research is needed to more fully explain the complex biochemistry behind these observations. Of note, inverse correlations between prenatal folate status and DNA methylation at differentially methylated loci have been identified in the other population studies including Hoyo *et al*.^34^ and Amarasekera *et al*.^12^

Although the health outcomes that have been related to folic acid supplementation involve target tissues such as the nervous system, we only had cord blood available for assessment of methylation. We do not know whether differential methylation at the sites that we observed in cord blood would be observed in relevant target tissues. While divergence in epigenetic patterns is critical for cell-type regulation, there is also evidence of similarities in patterns among some tissues^41^,^42^,^43^. We do not have data on methylation at older ages and thus the question of whether the differential methylation at these loci seen at birth in relation to maternal folate persists to later childhood would need to be addressed in future studies.

We measured folate using two different platforms in the two studies. Both are valid methods for the measurements of folate. Levels were reasonably similar although slightly higher in Generation R, which could reflect a difference in the platforms, differences in folate intake or the earlier timing of measurements in Generation R (∼12-week gestation in Generation R versus ∼18-week gestation in MoBa). Nonetheless, the top findings were consistent in both cohorts and thus robust to differences in measurement platforms. This may increase their generalizability to other populations.

One-carbon metabolism is a complex pathway with influences from multiple genetic, hormonal and environmental factors. Despite our attempt to account for other important dietary intake involved in one-carbon metabolism, other supplementation and genetic variants, residual confounding could still be present and influence the observed associations of folate levels in pregnancy with methylation at birth.

The MoBa and Generation R cohorts offer a unique opportunity to study the epigenetic effect of folic acid supplementation in the absence of food supply fortification. It is possible that results may differ in populations exposed to fortification.

We identified multiple novel genes not previously implicated in biological responses to folate. Many of the implicated genes have functional relevance to various developmental pathways, including the nervous system. Some of these are relevant not only to NTDs, the indication for maternal folic acid supplementation, but also to other developmental abnormalities that have not been previously associated with maternal folate levels. The associations between periconceptional folate and these conditions are difficult to study because the abnormalities are rare and both supplementation and fortification are now widespread. Other genes identified are implicated in conditions where concern exists about possible adverse effects of higher folate levels, such as breast cancer progression^16^. These findings may provide new insights into mechanisms for the associations between maternal folate status and health outcomes in the offspring. Given that food fortification programs have greatly increased the folate status of the population, greater understanding of the biological effects of this nutrient is important. The large number of novel genes identified using our genome-wide methylation approach may shed light on the protean effects of folate on human health.

## Methods

### Study populations

This analysis included participants of the Norwegian Mother and Child Cohort Study (MoBa)^44^,^45^ and participants of the Generation R Study from the Netherlands. The study populations and cohort-specific methods described below are more extensively detailed in the Supplementary Information (Supplementary Note). The MoBa participants were mother–offspring pairs from a substudy measuring maternal plasma folate during pregnancy^46^. The Generation R Study is a population-based prospective cohort study from fetal life onwards^47^,^48^. For this analysis, information on plasma folate and DNA methylation was available for 1,289 mothers and their children from the MoBa study (1,275 with complete covariate data) and 790 Caucasian mothers, and their children from the Generation R Study (713 with complete covariate data).

The MoBa study was approved by the Regional Committee for Ethics in Medical Research, the Norwegian Data Inspectorate and the Institutional Review Board of the National Institute of Environmental Health Sciences, USA, and written informed consent was provided by all mothers participating. The Generation R Study has been approved by the Medical Ethical Committee of the Erasmus MC, University Medical Center Rotterdam, Netherlands and written consent was obtained from participating parents of their children.

### Maternal plasma folate measurements

Both cohorts measured maternal plasma folate during pregnancy. For MoBa, maternal blood samples were drawn during pregnancy (median weeks gestation*=*18 weeks, 25–75th percentile=16–21 weeks) in EDTA-lined tubes, centrifuged within 30 min after collection and stored at 4 **°**C in the hospital where they were collected. Samples were then shipped overnight to the Biobank of MoBa at the Norwegian Institute of Public Health in Oslo. Upon receipt (1–2 days after blood collection), plasma was aliquoted onto polypropylene microtiter plates, sealed with heat-sealing foil sheets and stored at −80 **°**C. Plasma folate concentration was measured at Bevital AS (www.bevital.no) by microbiological assay, using a chloramphenicol-resistant strain of *Lactobacillus casei*^49^, which measures biologically active folate species, predominantly 5-methyl-tetrahydrofolate. The coefficient of variation (CV) for this assay corresponds to 4% within day and 5% between days, at population median.

For the Generation R cohort, venous blood samples were drawn at enrolment of the mothers in early pregnancy (median weeks gestation=12.9 weeks; 25–75th percentile*=*12.1–13.9 weeks) and stored at room temperature for a maximum of 3 h. Samples were transported to a laboratory facility of the regional laboratory in Rotterdam, Netherlands (Star-Medisch Diagnostisch Centrum) for additional processing and storage at −80 °C. The samples were analysed at the Department of Clinical Chemistry at the Erasmus MC, University Medical Center Rotterdam, Netherlands. After thawing, folate concentrations were analysed using an immunoelectrochemoluminence assay on the Architect System (Abbott Diagnostics BV). Between-run CVs for plasma measurements were 8.9% at 5.6 nmol l^−1^, 2.5% at 16.6 nmol l^−1^ and 1.5% at 33.6 nmol l^−1^ with an analytic range of 1.8–45.3 nmol l^−1^ for plasma folate.

### Covariates

Each cohort had information on maternal age, education and parity from questionnaires completed by the mother or from birth registry records. Maternal smoking during pregnancy was ascertained with questionnaires (both cohorts) and cotinine levels (MoBa). Plasma levels of vitamin B12, vitamin D and total homocysteine from samples taken during pregnancy were available for both cohorts. Mothers in both cohorts were genotyped for two SNPs in the (NAD(P)H) *MTHFR* gene, rs1801131 and rs1801133. Additional detail on these measurements is in the Supplementary Information (Supplementary Note).

### DNA methylation measurements

DNA was extracted from cord blood and bisulfite conversion performed (EZ-96 DNA Methylation kit, Zymo Research Corporation, Irvine, USA). Samples were processed with Illumina's Infinium HumanMethylation450 BeadChip (Illumina Inc., San Diego, USA) followed by cohort-specific laboratory quality control. Each cohort calculated the methylation betas, and normalized the betas using a published method^50^,^51^.

### Estimation of cell-type proportions

Both the MoBa and Generation R studies estimated cell-type proportion with the Houseman method^52^ as implemented in the *R minfi* package^53^ using the Reinius *et al*. data set for reference^54^. Cell-type correction was applied by including the six estimated cell-type proportions as covariates in cohort-specific statistical models.

### Cohort-specific statistical analyses

The cohort-specific statistical models were run independently. For each cohort, we used robust linear regression models in *R*^55^ to evaluate the association between natural log-transformed maternal plasma folate and cord blood DNA methylation for each probe while accounting for potential heteroskedasticity and/or influential outliers. Models were adjusted for maternal age, education, smoking during pregnancy, parity and for batch effects (adjustment for plate in Generation R, correction using *ComBat*^50^ in MoBa). Additional correction for study design was done in MoBa (whether the participant was in the MoBa1 or MoBa2 data set). Sex of the child was not expected to be associated with maternal plasma folate and was therefore not included as a covariate in the analyses. The adjustment variables were selected on *a priori* considerations and because they were also associated with maternal plasma folate levels at *P*<0.05.

### Meta-analysis

The probe-specific quality control resulted in 473,731 CpGs in the MoBa cohort and 436,013 CpGs in the Generation R cohort. The meta-analysis was limited to the 425,749 CpGs common to both cohorts. An additional 5,844 CpGs were excluded for having a SNP mapping to the last five nucleotides of the probe sequence and with a minor allele frequency ⩾5% in the CEU (Utah residents with North and Western European ancestry) population, curated by 1000G projects (http://www.1000genomes.org/, 06/2011 release, 87 individuals), HapMap project (http://hapmap.ncbi.nlm.nih.gov/, release 28, 8/2010, 174 individuals) and dbSNP (http://www.ncbi.nlm.nih.gov/projects/SNP/, build 134, 8/2011, 116 individuals). This left 419,905 CpGs for the final meta-analyses.

Fixed-effect meta-analysis weighted by the inverse of the variance was completed using *METAL*^56^. Multiple testing was accounted for by using the FDR procedure by Benjamini and Hochberg (BH)^57^. For each CpG, the resulting BH corrected *P* values are denoted by *P*_BH_. CpGs with *P*_BH_<0.05 were considered statistically significant. CpGs that were statistically significant based on the more stringent Bonferroni correction (uncorrected *P*<1.19 × 10^−7^ to account for 419,905 tests) were noted. We present the covariate-adjusted model without cell-type adjustment as the primary results. In the Supplementary Information, we present results additionally adjusted for cell type and results without covariate adjustment.

### Sensitivity analyses

We performed sensitivity analyses to assess whether the associations observed between folate and methylation might be explained by levels of vitamin B12, a dietary co-factor involved in regulating carbon unit bioavailability. Vitamin B12 is generally present in multivitamins that pregnant women in our studies may have taken in addition to, or in lieu of, separate folic acid supplements. Multivitamin supplements containing B12 typically contain other B vitamins including vitamin B6, which is also involved in one-carbon metabolism. Because mothers with higher folate levels may have higher intakes of other vitamin supplements not involved in the one-carbon metabolism pathway, or healthier diets in general, we also performed separate analyses adjusting for maternal plasma vitamin D levels during pregnancy. We also examined two SNPs in MTHFR involved in modulation of one-carbon metabolism: rs1801131 and rs1801133 (refs 14, 15). We evaluated the impact of adjustment for total homocysteine on the association between maternal plasma folate and DNA methylation in newborns. Finally, we examined whether the associations with methylation seen for maternal folate levels are also seen for newborn folate levels in a subset of 572 subjects in Generation R.

### Pathway analysis

To better understand the functional relationships between differentially methylated CpGs, we evaluated the FDR-significant CpGs with pathway analysis using three independent software programs. First, gene ontology analysis was performed using the IPA (www.ingenuity.com) based on the content version of 21249400 (release date: 22 September 2014). For a given category in IPA, Fisher's exact test was used to measure the probability that the category was randomly associated (*P*<0.05 defined as significantly enriched). Second, the NIAID's DAVID Bioinformatics Resources 6.7 (ref. 58) was used to analyse enrichments in main categories: biological process, cellular component, molecular function and KEGG pathway. Third, we used gene ontology enrichment analysis and visualization tool^59^ to identify the most informative terms that are significantly enriched.

### Methylation expression analysis

We evaluated the association between methylation and quantitative levels of gene expression for our top CpGs. We used messenger RNA gene expression and 450 K methylation data both from white blood cells from adults over 45 years of age in the Rotterdam Study, a population-based prospective cohort study in Rotterdam, the Netherlands. Among the 443 FDR-significant CpGs associated with folate, we were able to match 365 CpGs to a gene transcript in our gene expression data set within a region of 250-kb upstream or downstream of the CpG (total region 500 kb). We analysed the associations of these CpGs with expression levels of the corresponding gene transcripts.

## Additional information

**Accession codes:** The complete genome wide meta-analysis results file has been deposited in the database of genotypes and phenotypes (dbGaP) under the accession number phs001059.v1.p1. Access to individual-level Illumina HumanMethyl450 Beadchip data for the MoBa study dataset is available by application to the Norwegian Institute of Public Health using a form available on the English language portion of their website at http://www.fhi.no/eway/. Specific questions regarding MoBa data access can be directed to Wenche Nystad: Wenche.Nystad@fhi.no. Requests for access to the individual level data for the Generation R study can be directed to Liesbeth Duijts: l.duijts@erasmusmc.nl. For both studies the study management teams will verify with their local ethical committees that the applications are consistent with the consent provided. Applicants will need to obtain IRB approval or exemption from their local institutional review boards.

**How to cite this article**: Joubert, B. R. *et al*. Maternal plasma folate impacts differential DNA methylation in an epigenome-wide meta-analysis of newborns. *Nat. Commun.* 7:10577 doi: 10.1038/ncomms10577 (2016).

## Supplementary Material

Supplementary Information Supplementary Figures 1-3, Supplementary Tables 1-5, Supplementary Methods and Supplementary References

Supplementary Data 1 Meta-analysis of the association between maternal plasma folate during pregnancy and DNA methylation in newborns: CpGs statistically significant after FDR correction in covariate adjusted models, sorted by P value

Supplementary Data 2 Meta-analysis of the association between maternal plasma folate during pregnancy and DNA methylation in newborns: CpGs statistically significant after FDR correction in covariate adjusted models, sorted by chromosome and position. For comparison results from unadjusted and cell type adjusted meta-analyses are also presented

Supplementary Data 3 Sensitivity analysis results for the FDR-significant CpGs from the main covariate-adjusted model

## Figures and Tables

**Table 1:** Descriptive characteristics of the MoBa and Generation R study populations.

**Variable**	**Category**	**MoBa N (%)**	**Generation R N (%)**
Maternal plasma folate (nmol l^−1^)	Min–max	1.6–53.2	4.1–45.3
	Mean	11.9	20.3
	Median	9.1	19.6
	25–75th percentile	6.2–16.0	13.3–26.4
Log-transformed maternal plasma folate	Min–max	0.48–4.0	1.4–3.8
	Mean	2.3	2.9
	Median	2.2	3.0
	25–75th percentile	1.8–2.8	2.6–3.3
Maternal age (years)	Mean (s.d.)	29.9 (4.3)	31.5 (4.1)
Maternal education level	Less than secondary school	96 (7.5)	14 (1.8)
	Secondary school completion	415 (32.3)	267 (34.3)
	Some college or university	566 (44.1)	203 (26.0)
	4 Years or more of college/university	206 (16.1)	296 (37.9)
	Missing	6	10
Parity^*^	0	537 (41.7)	479 (60.7)
	1	511 (39.6)	240 (30.4)
	2	179 (13.9)	63 (8.0)
	3+	62 (4.8)	7 (0.9)
	Missing	0	1
Maternal smoking during pregnancy	No	1098 (85.2)	541 (75.6)
	Yes	191 (14.8)	175 (24.5)
	Missing	0	74

*N=*1,289 MoBa and *N=*790 Generation R participants with maternal plasma folate and newborn DNA methylation data. *N=*1,275 MoBa and *N=*713 Generation R participants with complete data were included in the adjusted models.

*Parity was categorized as ⩾1 versus 0 in the statistical models for the Generation R study.

**Table 2:** Selected loci with differential methylation in cord blood in relation to maternal folate.

**CHR**	**Position**	**CpG**	**Gene**	**Gene group**	**MoBa**			**Generation R**				**Meta-analysis**		
					**Coef**	**s.e.**	***P***	**Coef**	**s.e.**	***P***	**Weighted mean beta**	**Coef**	**s.e.**	***P***
7	126698829	cg15908975	*GRM8*	Body	−0.012	0.003	8.26E−06	−0.015	0.007	2.46E−02	0.54	−0.012	0.002	6.76E−07
7	126889015	cg18574254	*GRM8*	5′-UTR	−0.010	0.002	3.25E−06	−0.011	0.003	2.40E−04	0.84	−0.011	0.002	3.27E−09
10	91296252	cg22591480	*SLC16A12*	TSS1500	−0.009	0.002	1.96E−04	−0.008	0.003	2.33E−02	0.85	−0.008	0.002	1.34E−05
10	91296311	cg14920044	*SLC16A12*	TSS1500	−0.011	0.003	6.45E−05	−0.011	0.005	2.31E−02	0.78	−0.011	0.003	4.31E−06
11	132951838	cg24829292	*OPCML*	Body	0.008	0.003	9.45E−03	0.014	0.004	7.55E−05	0.44	0.010	0.002	6.60E−06
11	132951861	cg22629528	*OPCML*	Body	0.015	0.005	5.45E−03	0.031	0.009	4.54E−04	0.60	0.019	0.005	2.91E−05
11	132951950	cg26283170	*OPCML*	Body	0.006	0.002	9.46E−03	0.015	0.004	4.58E−05	0.54	0.009	0.002	1.30E−05
12	49689685	cg24804179	*PRPH*	Body	−0.007	0.002	3.73E−05	−0.007	0.004	8.72E−02	0.20	−0.007	0.002	8.05E−06
12	49690254	cg05775627	*PRPH*	Body	−0.006	0.002	3.61E−03	−0.007	0.002	7.87E−04	0.63	−0.007	0.002	1.01E−05
12	49692283	cg16010628	*PRPH*	3′-UTR	−0.006	0.001	8.58E−06	−0.002	0.003	4.51E−01	0.29	−0.005	0.001	1.73E−05
16	2866901	cg05635274	*PRSS21*	TSS1500	0.008	0.002	1.79E−04	0.013	0.005	5.05E−03	0.82	0.009	0.002	4.77E−06
16	2867051	cg02296564	*PRSS21*	TSS200	0.011	0.003	7.43E−04	0.011	0.004	2.63E−03	0.64	0.011	0.003	6.21E−06
16	2867434	cg22730830	*PRSS21*	Body	0.012	0.003	2.69E−04	0.016	0.005	4.02E−03	0.59	0.013	0.003	3.99E−06
16	2867446	cg01232511	*PRSS21*	Body	0.012	0.004	9.50E−04	0.020	0.006	2.32E−03	0.67	0.014	0.003	1.23E−05
17	35285205	cg10612259	*LHX1*		−0.009	0.003	1.91E−04	−0.015	0.004	5.54E−05	0.47	−0.011	0.002	9.10E−08
17	35285295	cg01965477	*LHX1*		−0.002	0.001	5.26E−04	−0.003	0.001	8.80E−03	0.06	−0.002	0.001	2.09E−05
19	1453909	cg11775595	*APC2*	Body	−0.017	0.003	8.04E−07	−0.010	0.005	3.57E−02	0.44	−0.015	0.003	1.64E−07
19	1456246	cg14907738	*APC2*	Body	−0.006	0.001	3.59E−05	−0.005	0.003	9.34E−02	0.22	−0.006	0.001	8.57E−06
19	1456337	cg27150178	*APC2*	Body	−0.009	0.002	6.01E−06	−0.007	0.003	3.00E−02	0.32	−0.009	0.002	5.81E−07
19	1456886	cg03165176	*APC2*	Body	−0.012	0.003	1.96E−04	−0.012	0.005	2.62E−02	0.58	−0.012	0.003	1.44E−05
19	1457211	cg14559388	*APC2*	Body	−0.003	0.001	3.49E−05	−0.002	0.001	4.52E−02	0.05	−0.003	0.001	4.98E−06
19	1465207	cg04624885	*APC2*	Body	−0.016	0.003	3.08E−07	−0.003	0.003	4.43E−01	0.66	−0.010	0.002	1.56E−05
19	1472936	cg19870717	*APC2*	3′-UTR	−0.010	0.002	1.81E−08	−0.006	0.003	4.46E−02	0.37	−0.009	0.002	4.64E−09
19	1473042	cg16613938	*APC2*	3′-UTR	−0.017	0.003	1.15E−07	−0.011	0.006	7.00E−02	0.71	−0.016	0.003	3.05E−08
19	1473179	cg23291200	*APC2*	3′-UTR	−0.011	0.002	7.81E−08	−0.008	0.003	3.92E−03	0.63	−0.010	0.002	1.72E−09
19	51415450	cg13793157	*KLK4*		−0.009	0.002	2.85E−04	−0.009	0.005	5.41E−02	0.53	−0.009	0.002	4.00E−05
19	51415452	cg10078829	*KLK4*		−0.007	0.002	1.89E−04	−0.007	0.003	3.57E−02	0.40	−0.007	0.002	1.84E−05

CHR, chromosome; Coef, regression coefficient from statistical model; gene, mapped or nearest gene (within 10 Mb) symbol using the UCSC database and Snipper software; gene group, gene region feature category (UCSC with verification); *P*, uncorrected *P* value from statistical model; UTR, untranslated region; weighted mean beta, average of the mean methylation beta values for MoBa and Generation R cohorts, weighted by cohort sample size.

Selection limited to genes with at least two CpGs at FDR significance that were prioritized for discussion.

Meta-analysis of results for 1,275 MoBa participants and 713 Generation R participants. Robust linear regression models adjusted for maternal age, maternal education, maternal sustained smoking during pregnancy, parity and batch. Results sorted by the chromosome and position of the CpG sites listed. For complete list of CpGs differentially methylated in relation to maternal folate and for results from meta-analysis models unadjusted for covariates and adjusted for covariates and cell type see Supplementary Data 1 (sorted by *P* value) and 2 (sorted by chromosome, position). Supplementary Data include columns for mapped and nearest gene for each CpG.
