# Supplementary material for: Maternal plasma folate impacts differential DNA methylation in an epigenome-wide meta-analysis of newborns
Source: Nat Commun. 2016 Feb 10;7:10577. doi: 10.1038/ncomms10577 (PMC4749955; doi:10.1038/ncomms10577)
Supplement: Supplementary Information — Supplementary Figures 1-3, Supplementary Tables 1-5, Supplementary Methods and Supplementary References [file ncomms10577-s1.pdf]

Maternal plasma folate impacts differential DNA methylation in an epigenome-wide meta-analysis of newborns

Supplementary Information

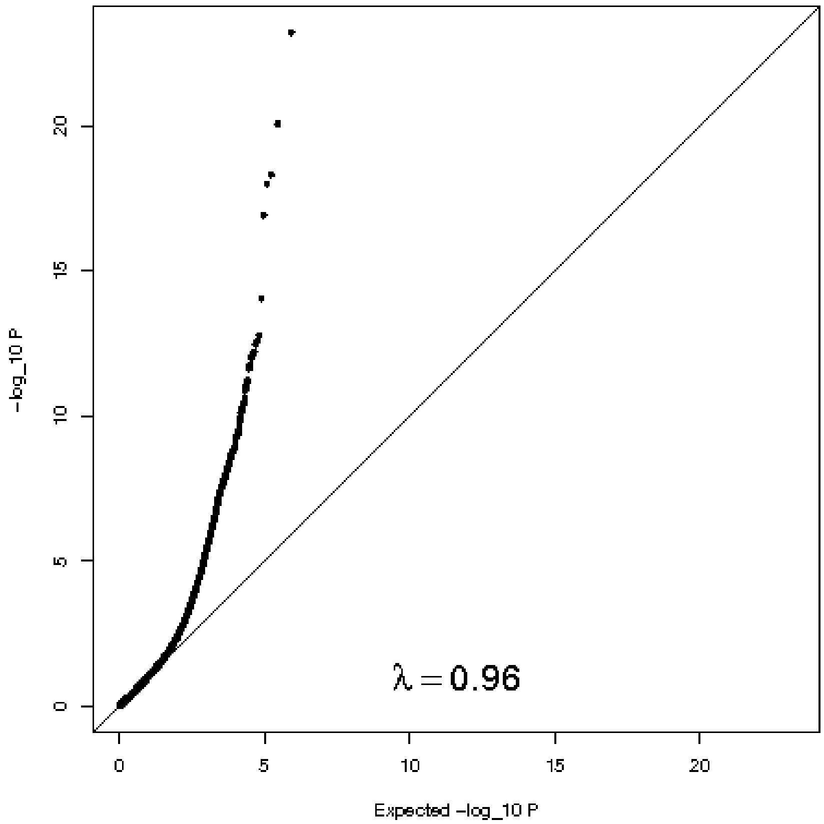

Supplementary Figure 1. Q-Q plot for the association between maternal plasma folate during pregnancy and DNA methylation in newborn cord blood, unadjusted for covariates. Meta-analysis results for MoBa (N=1,283) and Generation R (N=713) cohorts. The uncorrected –log10(P values) are plotted by expected –log10(P values). ʎ indicates the genomic inflation factor (lambda) for the model.

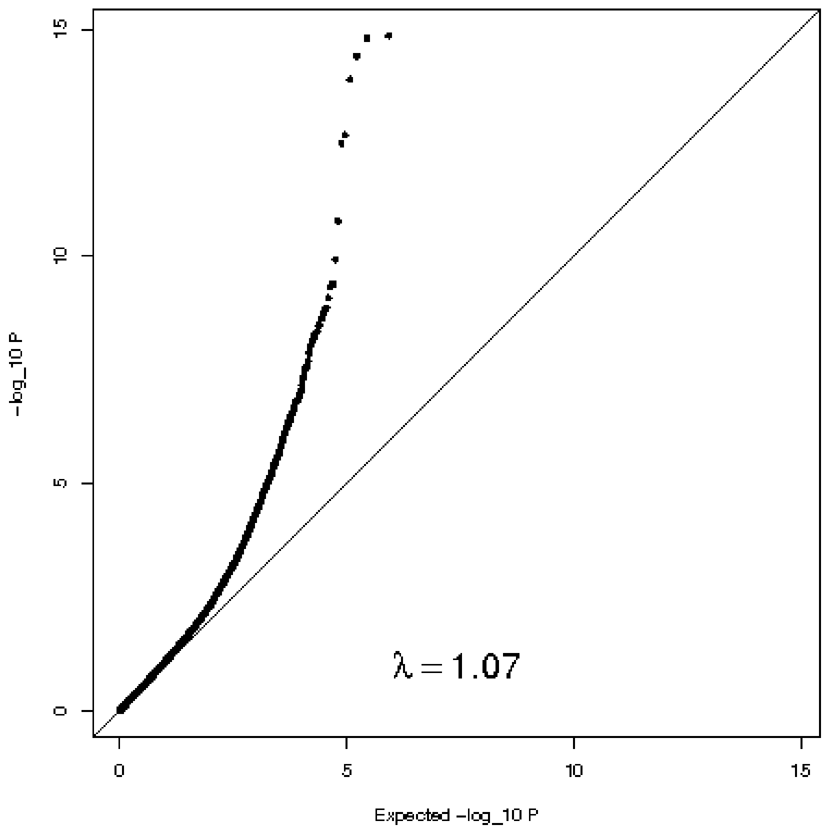

Supplementary Figure 2. Q-Q plot for the association between maternal plasma folate during pregnancy and DNA methylation in newborn cord blood, adjusted for covariates. Meta-analysis results for MoBa (N=1,283) and Generation R (N=713) cohorts. The uncorrected –log10(P values) are plotted by expected –log10(P values). ʎ indicates the genomic inflation factor (lambda) for the model.

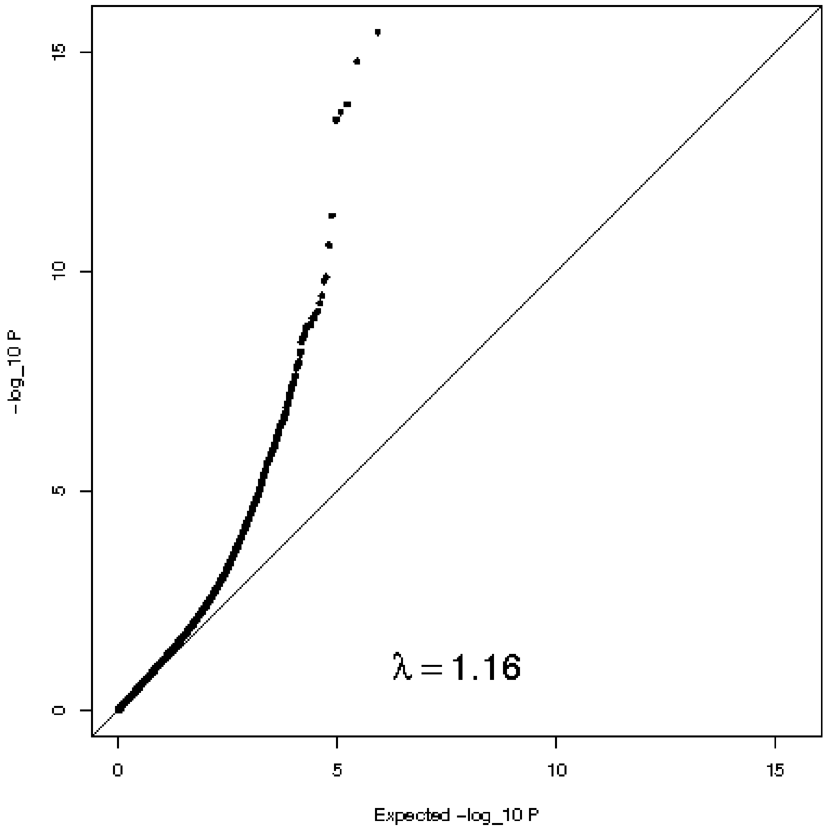

Supplementary Figure 3. Q-Q plot for the association between maternal plasma folate during pregnancy and DNA methylation in newborn cord blood, adjusted for covariates and cell type. Meta-analysis results for MoBa (N=1,283) and Generation R (N=713) cohorts. The uncorrected –log10(P values) are plotted by expected –log10(P values). λ indicates the genomic inflation factor (lambda) for the model.

Supplementary Table 1. Additional information on genes with significant CpGs after FDR correction in meta-analysis of the association between maternal plasma folate during pregnancy and DNA methylation in newborns

Gene Symbol	Gene Name	Entrez Gene UID	Synonyms
AATF	apoptosis antagonizing transcription factor	26574	*BFR2; CHE-1; DED; CHE1*
ABHD11	abhydrolase domain containing 11	83451	*PP1226; WBSCR21*
ADAMTS2	ADAM metallopeptidase with thrombospondin type 1 motif, 2	9509	*NPI; ADAMTS-3; ADAMTS-2; PCINP; ADAM-TS2; PNPI; PCPNI; PC; I-NP; PCI-NP*
ADCY8	adenylate cyclase 8 (brain)	114	*AC8; HBAC1; ADCY3*
ADRA2C	adrenoceptor alpha 2C	152	*ADRARL2; ALPHA2CAR; ADRA2RL2; ADRA2L2*
AJAP1	adherens junctions associated protein 1	55966	*MOT8; SHREW1; SHREW-1*
ALG10	ALG10, alpha-1,2-glucosyltransferase	84920	*DIE2; KCR1; ALG10A*
ALG10	ALG10, alpha-1,2-glucosyltransferase	84920	*DIE2; KCR1; ALG10A*
ALX3	ALX homeobox 3	257	*FND; FND1*
ANAPC7	anaphase promoting complex subunit 7	51434	*APC7*
ANKRD30BP3	ankyrin repeat domain 30B pseudogene 3	338579	*None*
AP3B2	adaptor-related protein complex 3, beta 2 subunit	8120	*NAPTB*
APC2	adenomatosis polyposis coli 2	10297	*APCL*
ARC	activity-regulated cytoskeleton-associated protein	23237	*Arg3.1*
ARRDC4	arrestin domain containing 4	91947	*None*
ASCL4	achaete-scute family bHLH transcription factor 4	121549	*bHLHa44; HASH4*
ATP5D	ATP synthase, H+ transporting, mitochondrial F1 complex, delta subunit	513	*None*
ATP8B3	ATPase, aminophospholipid transporter, class I, type 8B, member 3	148229	*ATPIK*
BAI2	brain-specific angiogenesis inhibitor 2	576	*None*
BCL7A	B-cell CLL/lymphoma 7A	605	*BCL7*
BOD1L2	biorientation of chromosomes in cell division 1-like 2	284257	*BOD1P; FAM44C*
BPGM	2,3-bisphosphoglycerate mutase	669	*DPGM*
BPIFA4P	BPI fold containing family A, member 4, pseudogene	317716	*BASE*
C11orf68	chromosome 11 open reading frame 68	83638	*P5326; BLES03*
C1orf35	chromosome 1 open reading frame 35	79169	*MMTAG2; hMMTAG2*
C3orf56	chromosome 3 open reading frame 56	285311	*FLJ40141*
C3P1	complement component 3 precursor pseudogene	388503	*CPLP*
C4orf17	chromosome 4 open reading frame 17	84103	*None*
C6orf52	chromosome 6 open reading frame 52	347744	*None*
CACNA1C	calcium channel, voltage-dependent, L type, alpha 1C subunit	775	*CCHL1A1; CaV1.2; CACN2; CACNL1A1; TS; CACH2; LQT8*
CACNG2	calcium channel, voltage-dependent, gamma subunit 2	10369	*MRD10*
CBLN2	cerebellin 2 precursor	147381	*None*
CCDC105	coiled-coil domain containing 105	126402	*None*
CCDC115	coiled-coil domain containing 115	84317	*ccp1*
CCDC166	coiled-coil domain containing 166	100130274	*None*
CCDC177	coiled-coil domain containing 177	56936	*PLPL; C14orf162*
CCDC36	coiled-coil domain containing 36	339834	*CT74*
CCDC64B	coiled-coil domain containing 64B	146439	*BICDR-2*
CCDC9	coiled-coil domain containing 9	26093	*None*
CCNG2	cyclin G2	901	*None*
CCT8L2	chaperonin containing TCP1, subunit 8 (theta)-like 2	150160	*CESK1*
CDC16	cell division cycle 16	8881	*APC6; CUT9; ANAPC6*
CDIPT	CDP-diacylglycerol--inositol 3-phosphatidyltransferase	10423	*PIS, PIS1*
CDO1	cysteine dioxygenase type 1	1036	*None*
CELF6	CUGBP, Elav-like family member 6	60677	*BRUNOL6*
CENPBD1	CENPB DNA-binding domains containing 1	92806	*None*
CES1	carboxylesterase 1	1066	*SES1; HMSE; hCE-1; CE-1; CEH; REH; CES2; HMSE1; ACAT; TGH; PCE-1*
CFB	complement factor B	629	*GBG; BF; PBF2; CFAB; BFD; CFBD; FB; ARMD14; FBI12; AHUS4; H2-Bf*

Gene Symbol	Gene Name	Entrez Gene UID	Synonyms
CFL1	cofilin 1 (non-muscle)	1072	*CFL*
CHFR	checkpoint with forkhead and ring finger domains, E3 ubiquitin protein ligase	55743	*RNF116; RNF196*
CHODL	chondrolectin	140578	*MT75; C21orf68; PRED12*
CHRD	chordin	8646	*None*
CKM	creatine kinase, muscle	1158	*CKMM; M-CK*
CLDN6	claudin 6	9074	*None*
CLMN	calmin (calponin-like, transmembrane)	79789	*None*
CLSTN2	calsyntenin 2	64084	*ALC-GAMMA; CDHR13; CSTN2; alcagamma; CS2*
CNPY1	canopy FGF signaling regulator 1	285888	*None*
CNST	consortin, connexin sorting protein	63882	*FLJ32001, PPP1R64*
CNTNAP5	contactin associated protein-like 5	129684	*caspr5*
COL13A1	collagen, type XIII, alpha 1	1305	*COLXIIIA1*
COPZ1	coatomer protein complex, subunit zeta 1	22818	*COPZ; CGI-120; zeta1-COP; zeta-COP; HSPC181*
CPLX1	complexin 1	10815	*CPX-I; CPX1*
CRHR1	corticotropin releasing hormone receptor 1	1394	*CRHR1L; CRH-R-1; CRF-R1; CRFR-1; CRF1; CRFR1; CRF-R-1; CRH-R1; CRF-R; CRHR*
CRIPAK	cysteine-rich PAK1 inhibitor	285464	*None*
CSMD1	CUB and Sushi multiple domains 1	64478	*PPP1R24*
CTRB2	chymotrypsinogen B2	440387	*None*
CUX2	cut-like homeobox 2	23316	*CDP2; CUTL2*
CWH43	cell wall biogenesis 43 C-terminal homolog (S. cerevisiae)	80157	*CWH43-C*
CYP4Z1	cytochrome P450, family 4, subfamily Z, polypeptide 1	199974	*CYP4A20*
DCC	DCC netrin 1 receptor	1630	*CRC18; CRCR1; NTN1R1; MRMV1; IGDCC1*
DDOST	dolichyl-diphosphooligosaccharide--protein glycosyltransferase subunit (non-catalytic)	1650	*CDG1R; OST48; OKSWcl45; OST; AGER1; WBP1*
DDX52	DEAD (Asp-Glu-Ala-Asp) box polypeptide 52	11056	*HUSSY19; ROK1*
DEFB115	defensin, beta 115	245929	*DEFB-15*
DEFB116	defensin, beta 116	245930	*DEFB-16*
DEFB125	defensin, beta 125	245938	*DEFB-25*
DIRC3	disrupted in renal carcinoma 3	729582	*None*
DKFZp434L192	uncharacterized protein DKFZp434L192	222029	*None*
DKFZp686K1684	uncharacterized LOC440034	440034	*None*
DKK1	dickkopf WNT signaling pathway inhibitor 1	22943	*SK; DKK-1*
DMBT1P1	deleted in malignant brain tumors 1 pseudogene 1	375940	*FLJ46361*
DRAP1	DR1-associated protein 1 (negative cofactor 2 alpha)	10589	*NC2-alpha*
DRD5	dopamine receptor D5	1816	*DRD1L2; DBDR; DRD1B*
DUXAP10	double homeobox A pseudogene 10	503639	*AK022914*
EARS2	glutamyl-tRNA synthetase 2, mitochondrial	124454	*COXPD12; MSE1*
EBF3	early B-cell factor 3	253738	*OE-2; COE3; O/E-2; EBF-3*
ECSIT	ECSIT signalling integrator	51295	*SITPEC*
EIF3L	eukaryotic translation initiation factor 3, subunit L	51386	*HSPC021; HSPC025; MSTP005; EIF3S11; EIF3EIP; EIF3S6IP*
ELP3	elongator acetyltransferase complex subunit 3	55140	*KAT9*
EMX2	empty spiracles homeobox 2	2018	*None*
EPS8L3	EPS8-like 3	79574	*EPS8R3*
ESM1	endothelial cell-specific molecule 1	11082	*endocan*
FAHD1	fumarylacetoacetate hydrolase domain containing 1	81889	*YISKL; C16orf36*
FAM188B	family with sequence similarity 188, member B	84182	*FLJ22374*
FAM193A	family with sequence similarity 193, member A	8603	*RES4-22; C4orf8*
FAM218A	family with sequence similarity 218, member A	152756	*C4orf39*
FAM24A	family with sequence similarity 24, member A	118670	*None*

Gene Symbol	Gene Name	Entrez Gene UID	Synonyms
FAM27L	family with sequence similarity 27-like	284123	*None*
FAM83H	family with sequence similarity 83, member H	286077	*AI3*
FBRSL1	fibrosin-like 1	57666	*None*
FBXL18	F-box and leucine-rich repeat protein 18	80028	*Fbl18*
FIGLA	folliculogenesis specific basic helix-loop-helix	344018	*BHLHC8; POF6; FIGALPHA*
FIS1	fission 1 (mitochondrial outer membrane) homolog (S. cerevisiae)	51024	*TTC11; CGI-135*
FLJ36000	uncharacterized FLJ36000	284124	*None*
FLYWCH2	FLYWCH family member 2	114984	*None*
FOXA2	forkhead box A2	3170	*TCF3B; HNF3B*
FOXN3	forkhead box N3	1112	*C14orf116; PRO1635; CHES1*
FRMD6	FERM domain containing 6	122786	*Willin; c14_5320; C14orf31; EX1*
FZD10	frizzled class receptor 10	11211	*FZ-10; FzE7; hFz10; CD350; Fz10*
FZD5	frizzled class receptor 5	7855	*HFZ5; C2orf31*
GABBR1	gamma-aminobutyric acid (GABA) B receptor, 1	2550	*GPRC3A; GABBR1-3; dJ271M21.1.2; dJ271M21.1.1; GABABR1; GB1*
GABRG3	gamma-aminobutyric acid (GABA) A receptor, gamma 3	2567	*None*
GJA3	gap junction protein, alpha 3, 46kDa	2700	*CTRCT14; CZP3; CX46*
GLI2	GLI family zinc finger 2	2736	*CJS; HPE9; THP2; THP1; PHS2*
GLI4	GLI family zinc finger 4	2738	*HKR4; ZNF928*
GLRX3	glutaredoxin 3	10539	*GLRX4; TXNL2; GRX3; TXNL3; PICOT; GRX4*
GNAI1	guanine nucleotide binding protein (G protein), alpha inhibiting activity polypeptide 1	2770	*None*
GNG7	guanine nucleotide binding protein (G protein), gamma 7	2788	*None*
GOLGA3	golgin A3	2802	*GCP170; MEA-2*
GRIN1	glutamate receptor, ionotropic, N-methyl D-aspartate 1	2902	*NMDA1; GluN1; MRD8; NMD-R1; NR1; NMDAR1*
GRIN2B	glutamate receptor, ionotropic, N-methyl D-aspartate 2B	2904	*MRD6; EIEE27; NR2B; hNR3; GluN2B; NMDAR2B*
GRM8	glutamate receptor, metabotropic 8	2918	*GPRC1H; GLUR8; mGlu8; MGLUR8*
GSG1L	GSG1-like	146395	*PRO19651*
GUSBP10	glucuronidase, beta pseudogene 10	642006	*None*
H19	H19, imprinted maternally expressed transcript (non-protein coding)	283120	*WT2; BWS; NCRNA00008; ASM1; D11S813E; PRO2605; LINC00008; ASM*
HES3	hes family bHLH transcription factor 3	390992	*bHLHb43*
HIST3H2BB	histone cluster 3, H2bb	128312	*H2Bb*
HSD17B7P2	hydroxysteroid (17-beta) dehydrogenase 7 pseudogene 2	158160	*Hsd17b_2; bA291L22.1; HSD17B7*
HSP90AA4P	heat shock protein 90kDa alpha (cytosolic), class A member 4, pseudogene	3323	*HSPCAL2; HSP90Ad*
IDH3B	isocitrate dehydrogenase 3 (NAD+) beta	3420	*RP46; H-IDHB*
IGDCC4	immunoglobulin superfamily, DCC subclass, member 4	57722	*NOPE; DDM36*
IHH	indian hedgehog	3549	*BDA1; HHG2*
INMT-FAM188B	INMT-FAM188B readthrough (NMD candidate)	100526825	*None*
INS-IGF2	INS-IGF2 readthrough	723961	*INSIGF*
IQCE	IQ motif containing E	23288	*1700028P05Rik*
KCNA1	potassium voltage-gated channel, shaker-related subfamily, member 1 (episodic ataxia with myokymia)	3736	*KV1.1; RBK1; HUK1; AEMK; MK1; EA1; MBK1; HBK1*
KCNJ9	potassium inwardly-rectifying channel, subfamily J, member 9	3765	*GIRK3; KIR3.3*
KCNQ1DN	KCNQ1 downstream neighbor (non-protein coding)	55539	*BWRT; HSA404617*
KCNV1	potassium channel, subfamily V, member 1	27012	*KCNB3; KV8.1; HNKA; KV2.3*
KIAA0125	KIAA0125	9834	*FAM30A; HSPC053; C14orf110*
KIF26A	kinesin family member 26A	26153	*None*
KIZ	kizuna centrosomal protein	55857	*Kizuna; C20orf19; HT013; RP69; PLK1S1; NCRNA00153*
KLHL18	kelch-like family member 18	23276	*None*
KLHL35	kelch-like family member 35	283212	*None*
KLK4	kallikrein-related peptidase 4	9622	*PSTS; EMSP; PRSS17; ARM1; KLK-L1; EMSP1; AI2A1; kallikrein*

Gene Symbol	Gene Name	Entrez Gene UID	Synonyms
KLKP1	kallikrein pseudogene 1	606293	*YKLK1; KLK31P; KARMA; KRSP1; PsiKLK1*
KREMEN2	kringle containing transmembrane protein 2	79412	*KRM2*
KRT28	keratin 28	162605	*KRT25D*
LAMP5	lysosomal-associated membrane protein family, member 5	24141	*BADLAMP; BAD-LAMP; UNC-43; LAMP-5; C20orf103*
LHX1	LIM homeobox 1	3975	*LIM1; LIM-1*
LINC00051	long intergenic non-protein coding RNA 51	619434	*NCRNA00051; C8orf43*
LINC00469	long intergenic non-protein coding RNA 469	283982	*C17orf54*
LINC00608	long intergenic non-protein coding RNA 608	51300	*None*
LINC01566	long intergenic non-protein coding RNA 1566	283914	*LOC283914*
LMO1	LIM domain only 1 (rhombotin 1)	4004	*TTG1; RHOM1; RBTN1*
LMX1B	LIM homeobox transcription factor 1, beta	4010	*NPS1; LMX1.2*
LOC100129636	uncharacterized LOC100129636	100129636	*None*
LOC100130700	uncharacterized LOC100130700	100130700	*None*
LOC100240728	uncharacterized LOC100240728	None	*BC036258*
LOC100294362	uncharacterized LOC100294362	100294362	*None*
LOC100506526	uncharacterized LOC100506526	None	*BC039381*
LOC283194	uncharacterized LOC283194	283194	*None*
LOC650226	ankyrin repeat domain 26 pseudogene	650226	*None*
LOC654342	lymphocyte-specific protein 1 pseudogene	654342	*None*
LOC728739	programmed cell death 2 pseudogene	728739	*None*
LRIF1	ligand dependent nuclear receptor interacting factor 1	55791	*C1orf103; RIF1*
LRIT2	leucine-rich repeat, immunoglobulin-like and transmembrane domains 2	340745	*LRRC22*
LRRC26	leucine rich repeat containing 26	389816	*bA350O14.10; CAPC*
MAP2K3	mitogen-activated protein kinase kinase 3	5606	*PRKMK3; MEK3; MAPKK3; SAPKK2; SAPKK-2; MKK3*
MAP3K10	mitogen-activated protein kinase kinase kinase 10	4294	*MLK2; MEKK10; MST*
MAP4K3	mitogen-activated protein kinase kinase kinase kinase 3	8491	*MEKKK3; MAPKKKK3; GLK; MEKKK; 3; RAB8IPL1*
MC3R	melanocortin 3 receptor	4159	*OQTL; OB20; MC3-R; MC3; BMIQ9*
MED24	mediator complex subunit 24	9862	*THRAP4; CRSP100; ARC100; CRSP4; TRAP100; DRIP100*
MGC72080	MGC72080 pseudogene	389538	*None*
MICU2	mitochondrial calcium uptake 2	221154	*EFHA1; 1110008L20Rik*
MIR205HG	MIR205 host gene	642587	*LINC00510*
MIR3147	microRNA 3147	100422939	*None*
MIR4283-1	microRNA 4283-1	100422917	*None*
MIR548G	microRNA 548g	100313938	*hsa-mir-548g; MIRN548G*
MIR9-3	microRNA 9-3	407051	*MIRN9-3; hsa-mir-9-3; miRNA9-3*
MKI67	marker of proliferation Ki-67	4288	*MIB-; KIA; MIB-1; PPP1R105*
MLLT1	myeloid/lymphoid or mixed-lineage leukemia (trithorax homolog, Drosophila); translocated to, 1	4298	*LTG19; YEATS1; ENL*
MMEL1	membrane metallo-endopeptidase-like 1	79258	*NL1; NEP2; NL2; SEP; MMEL2; NEPII*
MTRNR2L1	MT-RNR2-like 1	100462977	*HN1*
MTRNR2L7	MT-RNR2-like 7	100288485	*HN7*
NCAPH	non-SMC condensin I complex, subunit H	23397	*BRRN1; CAP-H*
NDUFA13	NADH dehydrogenase (ubiquinone) 1 alpha subcomplex, 13	51079	*B16.6, CDA016, CGI-39,GRIM-19, GRIM19*
NEUROG1	neurogenin 1	4762	*NEUROD3; AKA; Math4C; bHLHa6; ngn1*
NOXO1	NADPH oxidase organizer 1	124056	*SH3PXD5; P41NOX; SNX28; P41NOXC; P41NOXB; P41NOXA*
NPAS4	neuronal PAS domain protein 4	266743	*Le-PAS; PASD10; NXF; bHLHe79*
NR2E1	nuclear receptor subfamily 2, group E, member 1	7101	*XTLL; TLX; TLL*
NTF4	neurotrophin 4	4909	*NTF5; NT-4/5; NT5; NT4; GLC10; GLC1O; NT-5; NT-4*
OCA2	oculocutaneous albinism II	4948	*HCL3; SHEP1; BEY1; BEY2; EYCL; BOCA; P; BEY; PED; EYCL2; EYCL3; D15S12*
ODF3	outer dense fiber of sperm tails 3	113746	*CT135, hSHIPPO, SHIPPO1*
OGDH	oxoglutarate (alpha-ketoglutarate) dehydrogenase (lipoamide)	4967	*OGDC; E1k; AKGDH*

Gene Symbol	Gene Name	Entrez Gene UID	Synonyms
ONECUT1	one cut homeobox 1	3175	*HNF-6; HNF6A; HNF6*
OPCML	opioid binding protein/cell adhesion molecule-like	4978	*OBCAM; OPCM; IGLON1*
OR1A2	olfactory receptor, family 1, subfamily A, member 2	26189	*OR17-6*
OR1E1	olfactory receptor, family 1, subfamily E, member 1	8387	*OR1E8P; OR17-2; OST547; OR1E6; OR17-32; HGM071; OR1E5; OR1E9P; OR13-66*
OR1F1	olfactory receptor, family 1, subfamily F, member 1	4992	*OR16-88; OLFMF; OR16-36; OR16-37; OR16-90; OR1F13P; OR1F10; OR1F7; OR1F6; OR1F5; OR1F4; ORL1023; OR1F9; OR1F8; OR3-145; OR16-89*
OR2W1	olfactory receptor, family 2, subfamily W, member 1	26692	*hs6M1-15*
OR4K2	olfactory receptor, family 4, subfamily K, member 2	390431	*OR14-15*
OR4L1	olfactory receptor, family 4, subfamily L, member 1	122742	*OR14-28; OR4L2P*
OR7E37P	olfactory receptor, family 7, subfamily E, member 37 pseudogene	100506759	*OR7E48P; OST193; hg533*
ORAOV1	oral cancer overexpressed 1	220064	*TAOS1*
OSBPL7	oxysterol binding protein-like 7	114881	*ORP7*
OTOP1	otopetrin 1	133060	*None*
PABPC3	poly(A) binding protein, cytoplasmic 3	5042	*tPABP; PABPL3; PABP3*
PADI2	peptidyl arginine deiminase, type II	11240	*PDI2; PAD2; PAD-H19*
PAIP2B	poly(A) binding protein interacting protein 2B	400961	*None*
PANX2	pannexin 2	56666	*PX2; hPANX2*
PARP11	poly (ADP-ribose) polymerase family, member 11	57097	*MIB006; ARTD11; C12orf6*
PAX6	paired box 6	5080	*AN, D11S812E, WAGR*
PCDHB6	protocadherin beta 6	56130	*PCDH-BETA6*
PCP2	Purkinje cell protein 2	126006	*GPSM4*
PCSK9	proprotein convertase subtilisin/kexin type 9	255738	*PC9; FH3; HCHOLA3; NARC1; LDLCQ1; NARC-1*
PLEKHD1	pleckstrin homology domain containing, family D (with coiled-coil domains) member 1	400224	*UPF0639*
PLXNA1	plexin A1	5361	*NOV; PLEXIN-A1; PLXN1; NOVP*
POLR3A	polymerase (RNA) III (DNA directed) polypeptide A, 155kDa	11128	*RPC155; HLD7; hRPC155; RPC1; ADDH*
POTEA	POTE ankyrin domain family, member A	340441	*POTE-8; CT104.3; A26A1; POTE8*
POTEH	POTE ankyrin domain family, member H	23784	*CT104.7; A26C3; POTE22; ACTBL1*
PPIE	peptidylprolyl isomerase E (cyclophilin E)	10450	*CYP-33; CYP33*
PPP1R3G	protein phosphatase 1, regulatory subunit 3G	648791	*None*
PRDM1	PR domain containing 1, with ZNF domain	639	*PRDI-BF1; BLIMP1*
PRDM13	PR domain containing 13	59336	*PFM10; MU-MB-20.220*
PRG3	proteoglycan 3	10394	*MBPH; MBP2*
PRPH	peripherin	5630	*NEF4; PRPH1*
PRR14L	proline rich 14-like	253143	*C22orf30*
PRR18	proline rich 18	285800	*None*
PRR30	proline rich 30	339779	*C2orf53*
PRR30	proline rich 30	39779	*MGC44505*
PRSS21	protease, serine, 21 (testisin)	10942	*TEST1; ESP1; TESTISIN; ESP-1*
PSPH	phosphoserine phosphatase	5723	*PSP*
PTGER3	prostaglandin E receptor 3 (subtype EP3)	5733	*EP3-IV; PGE2-R; EP3e; EP3; EP3-III; EP3-I; EP3-II*
PTHLH	parathyroid hormone-like hormone	5744	*PTHR; BDE2; PTHRP; HHM; PLP*
PTPRH	protein tyrosine phosphatase, receptor type, H	5794	*R-PTP-H; SAP1*
PTPRU	protein tyrosine phosphatase, receptor type, U	10076	*FMI, hPTP-J, PCP-2, PTP, PTPRO*
PTX4	pentraxin 4, long	390667	*C16orf38*
PXDC1	PX domain containing 1	221749	*C6orf145*
QTRT1	queuine tRNA-ribosyltransferase 1	81890	*TGUT; TGT; FP3235*
RASA3	RAS p21 protein activator 3	22821	*GAPIII; GAP1IP4BP*
REEP1	receptor accessory protein 1	65055	*SPG31; C2orf23; HMN5B*
RGCC	regulator of cell cycle	28984	*RGC-32; bA157L14.2; C13orf15; RGC32*
RNF180	ring finger protein 180	285671	*RINES*

Gene Symbol	Gene Name	Entrez Gene UID	Synonyms
RNF207	ring finger protein 207	388591	*C1orf188*
ROBO3	roundabout, axon guidance receptor, homolog 3 (Drosophila)	64221	*HGPS; RIG1; RBIG1; HGPPS*
ROPN1	rhophilin associated tail protein 1	54763	*ROPN1A; RHPNAP1; ODF6; ropporin; CT91*
RPA1	replication protein A1, 70kDa	6117	*HSSB; RF-A; RP-A; REPA1; MST075; RPA70*
SALL1	spalt-like transcription factor 1	6299	*HSAL1; ZNF794; TBS; HEL-S-89; Sal-1*
SARM1	sterile alpha and TIR motif containing 1	23098	*MyD88-5; SAMD2; SARM*
SCGB1C1	secretoglobin, family 1C, member 1	147199	*RYD5*
SCGB1D4	secretoglobin, family 1D, member 4	404552	*None*
SEC14L3	SEC14-like 3 (S. cerevisiae)	266629	*TAP2*
SELV	selenoprotein V	348303	*None*
SFSWAP	splicing factor, suppressor of white-apricot family	6433	*SWAP; SFRS8*
SLC16A12	solute carrier family 16, member 12	387700	*MCT12; CJMG*
SLC39A3	solute carrier family 39 (zinc transporter), member 3	29985	*ZIP3; ZIP-3*
SLC39A7	solute carrier family 39 (zinc transporter), member 7	7922	*HKE4; RING5; H2-KE4; D6S115E; D6S2244E; KE4; ZIP7*
SLCO2B1	solute carrier organic anion transporter family, member 2B1	11309	*OATP2B1; OATP-B; SLC21A9; OATPB*
SNAP47	synaptosomal-associated protein, 47kDa	116841	*SVAP1; HEL170; SNAP-47; C1orf142*
SNORD115-11	small nucleolar RNA, C/D box 115-11	100033448	*HBII-52-11*
SNORD115-15	small nucleolar RNA, C/D box 115-15	100033453	*HBII-52-15*
SNORD116-11	small nucleolar RNA, C/D box 116-11	100033423	*HBII-85-11*
SNORD116-12	small nucleolar RNA, C/D box 116-12	100033424	*HBII-85-12*
SNORD116-17	small nucleolar RNA, C/D box 116-17	100033429	*HBII-85-17*
SNORD116-18	small nucleolar RNA, C/D box 116-18	100033430	*HBII-85-18*
SNRPN	small nuclear ribonucleoprotein polypeptide N	6638	*SMN; PWCR; SM-D; sm-N; RT-LI; HCERN3; SNRNP-N; SNURF-SNRPN*
SNX32	sorting nexin 32	254122	*SNX6B*
SOX14	SRY (sex determining region Y)-box 14	8403	*SOX28*
SPRY2	sprouty homolog 2 (Drosophila)	10253	*hSPRY2*
STMN2	stathmin 2	11075	*SCG10; SCGN10*
SUMF1	sulfatase modifying factor 1	285362	*FGE; UNQ3037; AAPA3037*
SYT5	synaptotagmin V	6861	*None*
TBX3	T-box 3	6926	*TBX3-ISO; UMS; XHL*
TCTE1	t-complex-associated-testis-expressed 1	202500	*D6S46; FAP155*
TEKT2	tektin 2 (testicular)	27285	*h-tektin-t; TEKTB1; TEKTIN-T*
TENM4	teneurin transmembrane protein 4	26011	*TNM4; ODZ4; Doc4; Ten-M4*
TENM4	teneurin transmembrane protein 4	26011	*ODZ4*
TFB2M	transcription factor B2, mitochondrial	64216	*Hkp1; mtTFB2*
THBS2	thrombospondin 2	7058	*TSP2*
THSD1P1	thrombospondin, type I, domain containing 1 pseudogene 1	374500	*THSD1P*
TMEM138	transmembrane protein 138	51524	*HSPC196*
TMEM18	transmembrane protein 18	129787	*None*
TMEM200C	transmembrane protein 200C	645369	*TTMA*
TNS3	tensin 3	64759	*TEM6; TENS1*
TPTE	transmembrane phosphatase with tensin homology	7179	*PTEN2; CT44*
TRABD2B	TraB domain containing 2B	388630	*TIKI2*
TRIM41	tripartite motif containing 41	90933	*RINCK*
TRIM61	tripartite motif containing 61	391712	*RNF35*
TRR-CCT5-1	transfer RNA-Arg (CCT) 5-1	100189270	*TRNAR23*
TSSK6	testis-specific serine kinase 6	83983	*SSTK; TSSK4; FKSG82; CT72*
TTC34	tetratricopeptide repeat domain 34	100287898	*None*
TTYH3	tweety family member 3	80727	*None*
UBE2MP1	ubiquitin-conjugating enzyme E2M pseudogene 1	606551	*None*
ULBP2	UL16 binding protein 2	80328	*RAET1H; ALCAN-alpha; NKG2DL2; N2DL2*

Gene Symbol	Gene Name	Entrez Gene UID	Synonyms
UNC93B1	unc-93 homolog B1 (C. elegans)	81622	UNC93B; Unc-93B1; IIAE1; UNC93
USP29	ubiquitin specific peptidase 29	57663	HOM-TES-84/86
WDR24	WD repeat domain 24	84219	C16orf21; JFP7
WDR37	WD repeat domain 37	22884	None
WNT3A	wingless-type MMTV integration site family, member 3A	89780	None
WSB1	WD repeat and SOCS box containing 1	26118	WSB-1; SWIP1
YBX2	Y box binding protein 2	51087	DBPC; CSDA3; CONTRIN; MSY2
ZACN	zinc activated ligand-gated ion channel	353174	LGICZ1; L2; ZAC; LGICZ; ZAC1
ZBTB32	zinc finger and BTB domain containing 32	27033	TZFP; FAXF; FAZF; ZNF538; Rog
ZC3H14	zinc finger CCCH-type containing 14	79882	UKp68; MSUT-2; SUT2; NY-REN-37
ZNF248	zinc finger protein 248	57209	bA162G10.3
ZNF267	zinc finger protein 267	10308	HZF2
ZNF502	zinc finger protein 502	91392	None
ZNF569	zinc finger protein 569	148266	ZAP1; ZNF
ZNF570	zinc finger protein 570	148268	FLJ30791
ZNF595	zinc finger protein 595	152687	None
ZNF596	zinc finger protein 596	169270	None
ZNF613	zinc finger protein 613	79898	None
ZNF638	zinc finger protein 638	27332	MGC26130, NP220, Zfp638
ZNF718	zinc finger protein 718	255403	FLJ90036
ZNF727	zinc finger protein 727	442319	ZNF727P
ZNF733P	zinc finger protein 733, pseudogene	643955	ZNF733
ZNF735	zinc finger protein 735	730291	ZNF735P
ZNF860	zinc finger protein 860	344787	None
ZSWIM4	zinc finger, SWIM-type containing 4	65249	None
ZZEF1	zinc finger, ZZ-type with EF-hand domain 1	23140	ZZZ4
TRABD2B	TraB domain containing 2B	388630	TIKI2
TRIM41	tripartite motif containing 41	90933	RINCK
TRIM61	tripartite motif containing 61	391712	RNF35
TRR-CCT5-1	transfer RNA-Arg (CCT) 5-1	100189270	TRNAR23
TSSK6	testis-specific serine kinase 6	83983	SSTK; TSSK4; FKSG82; CT72
TTC34	tetratricopeptide repeat domain 34	100287898	None
TTYH3	tweety family member 3	80727	None
UBE2MP1	ubiquitin-conjugating enzyme E2M pseudogene 1	606551	None
ULBP2	UL16 binding protein 2	80328	RAET1H; ALCAN-alpha; NKG2DL2; N2DL2
UNC93B1	unc-93 homolog B1 (C. elegans)	81622	UNC93B; Unc-93B1; IIAE1; UNC93
USP29	ubiquitin specific peptidase 29	57663	HOM-TES-84/86
WDR24	WD repeat domain 24	84219	C16orf21; JFP7
WDR37	WD repeat domain 37	22884	None
WNT3A	wingless-type MMTV integration site family, member 3A	89780	None
WSB1	WD repeat and SOCS box containing 1	26118	WSB-1; SWIP1
YBX2	Y box binding protein 2	51087	DBPC; CSDA3; CONTRIN; MSY2
ZACN	zinc activated ligand-gated ion channel	353174	LGICZ1; L2; ZAC; LGICZ; ZAC1
ZBTB32	zinc finger and BTB domain containing 32	27033	TZFP; FAXF; FAZF; ZNF538; Rog
ZC3H14	zinc finger CCCH-type containing 14	79882	UKp68; MSUT-2; SUT2; NY-REN-37
ZNF248	zinc finger protein 248	57209	bA162G10.3
ZNF267	zinc finger protein 267	10308	HZF2
ZNF502	zinc finger protein 502	91392	None
ZNF569	zinc finger protein 569	148266	ZAP1; ZNF
ZNF570	zinc finger protein 570	148268	FLJ30791
ZNF595	zinc finger protein 595	152687	None

Gene Symbol	Gene Name	Entrez Gene UID	Synonyms
ZNF596	zinc finger protein 596	169270	*None*
ZNF613	zinc finger protein 613	79898	*None*
ZNF638	zinc finger protein 638	27332	*MGC26130, NP220, Zfp638*
ZNF718	zinc finger protein 718	255403	*FLJ90036*
ZNF727	zinc finger protein 727	442319	*ZNF727P*
ZNF733P	zinc finger protein 733, pseudogene	643955	*ZNF733*
ZNF735	zinc finger protein 735	730291	*ZNF735P*
ZNF860	zinc finger protein 860	344787	*None*
ZSWIM4	zinc finger, SWIM-type containing 4	65249	*None*
ZZEF1	zinc finger, ZZ-type with EF-hand domain 1	23140	*ZZZ4*

Additional information provided using Snipper software for nearest genes that were validated in the UCSC database.

Supplementary Table 2. Biological processes considered statistically significant in the DAVID pathway analysis

Biological process	Count	%	Fold Enrichment	P Value	Benjamini
cell development	17	8.30	2.6	0.0008	0.28
embryonic morphogenesis	11	5.30	3.5	0.0012	0.21
pattern specification process	10	4.90	3.7	0.0016	0.19
regulation of multicellular organismal process	20	9.70	2.1	0.0030	0.26
cell-cell signaling	15	7.30	2.4	0.0032	0.22
regionalization	8	3.90	4.0	0.0040	0.23
forebrain development	7	3.40	4.5	0.0046	0.23
neural tube development	5	2.40	7.2	0.0051	0.22
embryonic development	14	6.80	2.4	0.0052	0.21
behavioral interaction between organisms	4	1.90	11.1	0.0053	0.19
embryonic development ending in birth or egg hatching	10	4.90	2.9	0.0071	0.23
negative regulation of molecular function	10	4.90	2.9	0.0071	0.23
formation of primary germ layer	4	1.90	9.8	0.0077	0.23
osteoblast differentiation	4	1.90	9.3	0.0089	0.24
neurological system process	22	10.70	1.8	0.0110	0.26
pallium development	4	1.90	8.5	0.0110	0.26
ion transport	16	7.80	2.0	0.0110	0.25
negative regulation of cellular metabolic process	15	7.30	2.0	0.0150	0.29
positive regulation of cellular metabolic process	17	8.30	1.9	0.0170	0.31
anatomical structure morphogenesis	21	10.20	1.7	0.0180	0.32
negative regulation of cell differentiation	7	3.40	3.2	0.0230	0.37
hindbrain development	4	1.90	6.5	0.0230	0.36
cell differentiation	26	12.60	1.5	0.0240	0.35
regulation of nitrogen compound metabolic process	40	19.40	1.4	0.0240	0.34
positive regulation of metabolic process	17	8.30	1.8	0.0250	0.34
tube development	7	3.40	3.1	0.0250	0.33
regulation of primary metabolic process	45	21.80	1.3	0.0250	0.32
response to organic nitrogen	4	1.90	6.2	0.0260	0.32
system development	34	16.50	1.4	0.0270	0.31
learning or memory	5	2.40	4.4	0.0270	0.31
organ development	27	13.10	1.5	0.0270	0.30
negative regulation of metabolic process	15	7.30	1.9	0.0280	0.30
telencephalon development	4	1.90	6.0	0.0280	0.30
visual learning	3	1.50	10.8	0.0310	0.31
oxygen and reactive oxygen species metabolic process	4	1.90	5.8	0.0310	0.30
negative regulation of biosynthetic process	12	5.80	2.0	0.0320	0.31
negative regulation of macromolecule metabolic process	14	6.80	1.9	0.0360	0.33
bone development	5	2.40	4.0	0.0370	0.33
regulation of cellular metabolic process	46	22.30	1.3	0.0370	0.32
visual behavior	3	1.50	9.4	0.0390	0.33
morphogenesis of a branching structure	4	1.90	5.3	0.0400	0.33
tube morphogenesis	5	2.40	3.8	0.0410	0.33
regulation of biosynthetic process	40	19.40	1.3	0.0450	0.35
regulation of nervous system development	6	2.90	3.0	0.0460	0.35
negative regulation of developmental process	7	3.40	2.6	0.0490	0.36
regulation of catalytic activity	15	7.30	1.7	0.0490	0.36
defense response	12	5.80	1.9	0.0490	0.35
**Cellular component**	**Count**	**%**	**Fold Enrichment**	**P value**	**Benjamini**
postsynaptic membrane	7	3.40	5.2	0.0022	0.21
plasma membrane part	33	16.00	1.5	0.0150	0.55
intrinsic to membrane	68	33.00	1.2	0.0190	0.49
plasma membrane	50	24.30	1.3	0.0190	0.40
membrane part	79	38.30	1.2	0.0200	0.35
postsynaptic density	4	1.90	5.7	0.0330	0.45
membrane	84	40.80	1.2	0.0400	0.46
**Molecular function**	**Count**	**%**	**Fold Enrichment**	**P value**	**Benjamini**
passive transmembrane transporter activity	12	5.80	2.9	0.0031	0.23
substrate-specific transmembrane transporter activity	17	8.30	2.0	0.0092	0.32
receptor activity	27	13.10	A	0.0460	0.73
**Pathway**	**Count**	**%**	**Fold Enrichment**	**P value**	**Benjamini**
Neuroactive ligand-receptor interaction	9	4.40	3.3	0.0046	0.27
Amyotrophic lateral sclerosis (ALS)	4	1.90	7.1	0.0170	0.45
Long-term potentiation	4	1.90	5.5	0.0330	0.54

Supplementary Table 3. Top results from Ingenuity Pathway Analysis

Top Canonical Pathways	P value	Ratio	Molecules
Glutamate Receptor Signaling	0.002	6.25E-02	GRIN2B,GRIN1,GRM8,GNG7
Gαs Signaling	0.003	4.42E-02	MC3R,DRD5,ADCY8,CRHR1,GNG7
cAMP-mediated signaling	0.003	3.14E-02	GRM8,MC3R,PTGER3,DRD5,GABBR1,ADCY8,CRHR1
Dopamine-DARPP32 Feedback in cAMP Signaling	0.004	3.43E-02	GRIN2B,GRIN1,KCNJ9,CACNA1C,DRD5,ADCY8
Gαi Signaling	0.004	4.07E-02	GRM8,PTGER3,GABBR1,ADCY8,GNG7
Synaptic Long Term Potentiation	0.005	3.94E-02	GRIN2B,GRIN1,GRM8,CACNA1C,ADCY8
G-Protein Coupled Receptor Signaling	0.008	2.65E-02	GRM8,MC3R,PTGER3,DRD5,GABBR1,ADCY8,CRHR1
IL-1 Signaling	0.009	4.26E-02	ECSIT,MAP2K3,ADCY8,GNG7
Amyotrophic Lateral Sclerosis Signaling	0.014	3.74E-02	PRPH,GRIN2B,GRIN1,CACNA1C
Wnt/β-catenin Signaling	0.016	2.94E-02	WNT3A,APC2,FZD5,KREMEN2,DKK1
Colorectal Cancer Metastasis Signaling	0.018	2.49E-02	WNT3A,PTGER3,DCC,FZD5,ADCY8,GNG7
CREB Signaling in Neurons	0.019	2.79E-02	GRIN2B,GRIN1,GRM8,ADCY8,GNG7
Maturity Onset Diabetes of Young (MODY) Signaling	0.021	7.69E-02	FOXA2,CACNA1C
Basal Cell Carcinoma Signaling	0.025	4.11E-02	WNT3A,APC2,FZD5
GABA Receptor Signaling	0.025	4.11E-02	GABRG3,GABBR1,ADCY8
Role of Wnt/GSK-3β Signaling in the Pathogenesis of Influenza	0.031	3.80E-02	WNT3A,APC2,FZD5
Circadian Rhythm Signaling	0.036	5.71E-02	GRIN2B,GRIN1
Role of Osteoblasts, Osteoclasts and Chondrocytes in Rheumatoid Arthritis	0.046	2.22E-02	WNT3A,APC2,FZD5,MAP2K3,DKK1
Transcriptional Regulatory Network in Embryonic Stem Cells	0.047	5.00E-02	NEUROG1,PAX6
TCA Cycle II (Eukaryotic)	0.049	4.88E-02	OGDH,IDH3B
Cardiac Hypertrophy Signaling	0.050	2.16E-02	MAP3K10,CACNA1C,MAP2K3,ADCY8,GNG7
**Top Upstream Regulators**	**Molecule Type**	**P value of overlap**	**Target molecules in dataset**
EPHB2	kinase	1.83E-05	GRIN1,GRIN2B,STMN2
BAIAP2	kinase	1.98E-04	GRIN1,GRIN2B
NTRK3	kinase	3.94E-04	GRIN1,GRIN2B
GABBR2	g-protein coupled receptor	6.53E-04	ARC,GABBR1
ifenprodil	chemical drug	6.53E-04	GRIN1,GRIN2B
ganciclovir	chemical drug	9.74E-04	ARC,THBS2
flupenthixol	chemical drug	1.36E-03	GRIN1,GRIN2B
aroclor 1221	chemical toxicant	1.80E-03	GRIN2B,MC3R
ASF1A	other	2.30E-03	FOXA2,PAX6
SUZ12	enzyme	2.76E-03	KCNA1,PAX6,TBX3,TNS3,WNT3A
**Top Diseases and Disorders**	**Minimum P value**	**# Molecules**	
Cancer	2.50E-07	160	
Neurological Disease	2.60E+01	32	
Connective Tissue Disorders	5.33E-04	13	
Dental Disease	5.33E-04	5	
Developmental Disorder	5.33E-04	28	
**Top Molecular and Cellular Functions**	**Minimum P value**	**# Molecules**	
Cellular growth and proliferation	7.35E-05	11	
Cell-to-cell signaling and interaction	1.13E-04	26	
Cellular development	1.43E-04	31	
Cellular assembly and organization	3.89E-04	21	
Cell signaling	3.95E-04	18	
**Top Toxicity Lists**	**P value**	**Ratio**	**Molecules**
Increases Renal Damage	2.95E-02	3.85E-02	CFB,RGCC,MAP2K3
Decreases Permeability Transition of Mitochondria and Mitochondrial Membrane	5.89E-02	1.43E-01	OGDH
Mitochondrial Dysfunction	6.61E-02	2.27E-02	FIS1,ATP5D,OGDH,NDUFA13
Glutathione Depletion - CYP Induction and Reactive Metabolites	9.77E-02	8.33E-02	CFB
PPARα/RXRα Activation	2.09E-01	1.64E-02	MAP2K3,ADCY8,MED24

Supplementary Table 4. Functional annotation results from GORILLA

GO term	Description	P value	FDR q-value	Enrichment (N, B, n, b)*	Genes
GO:0007268	synaptic transmission	1.24E-05	1.61E-01	3.55 (18004,443,183,16)	
					GNG7 - guanine nucleotide binding protein (g protein), gamma 7
					GRIN1 - glutamate receptor, ionotropic, n-methyl d-aspartate 1
					CACNG2 - calcium channel, voltage-dependent, gamma subunit 2
					KCNA1 - potassium voltage-gated channel, shaker-related subfamily, member 1 (episodic ataxia with myokymia)
					GRIN2B - glutamate receptor, ionotropic, n-methyl d-aspartate 2b
					GABBR1 - gamma-aminobutyric acid (gaba) b receptor, 1
					DRD5 - dopamine receptor d5
					CPLX1 - complexin 1
					KCNJ9 - potassium inwardly-rectifying channel, subfamily j, member 9
					SYT5 - synaptotagmin v
					GABRG3 - gamma-aminobutyric acid (gaba) a receptor, gamma 3
					KCNV1 - potassium channel, subfamily v, member 1
					PANX2 - pannexin 2
					ADCY8 - adenylate cyclase 8 (brain)
					PCDHB6 - protocadherin beta 6
					CACNA1C - calcium channel, voltage-dependent, l type, alpha 1c subunit
GO:0007267	cell-cell signaling	4.67E-05	3.04E-01	2.73 (18004,721,183,20)	
					NTF4 - neurotrophin 4
					CACNG2 - calcium channel, voltage-dependent, gamma subunit 2
					GNG7 - guanine nucleotide binding protein (g protein), gamma 7
					GRIN1 - glutamate receptor, ionotropic, n-methyl d-aspartate 1
					KCNA1 - potassium voltage-gated channel, shaker-related subfamily, member 1 (episodic ataxia with myokymia)
					GRIN2B - glutamate receptor, ionotropic, n-methyl d-aspartate 2b
					GABBR1 - gamma-aminobutyric acid (gaba) b receptor, 1
					DRD5 - dopamine receptor d5
					CPLX1 - complexin 1
					GJA3 - gap junction protein, alpha 3, 46kda
					KCNJ9 - potassium inwardly-rectifying channel, subfamily j, member 9
					SYT5 - synaptotagmin v
					GABRG3 - gamma-aminobutyric acid (gaba) a receptor, gamma 3
					KCNV1 - potassium channel, subfamily v, member 1
					PANX2 - pannexin 2
					PTHLH - parathyroid hormone-like hormone
					ADCY8 - adenylate cyclase 8 (brain)
					PCDHB6 - protocadherin beta 6
					CACNA1C - calcium channel, voltage-dependent, l type, alpha 1c subunit
					IHH - indian hedgehog
GO:0030817	regulation of cAMP biosynthetic process	6.34E-05	2.75E-01	7.03 (18004,98,183,7)	
					GRM8 - glutamate receptor, metabotropic 8
					GNG7 - guanine nucleotide binding protein (g protein), gamma 7
					CRHR1 - corticotropin releasing hormone receptor 1
					GABBR1 - gamma-aminobutyric acid (gaba) b receptor, 1
					DRD5 - dopamine receptor d5
					PTHLH - parathyroid hormone-like hormone
					MC3R - melanocortin 3 receptor
GO:0044708	single-organism behavior	6.43E-05	2.09E-01	3.64 (18004,351,183,13)	
					GNG7 - guanine nucleotide binding protein (g protein), gamma 7
					NTF4 - neurotrophin 4

GO term	Description	P value	FDR q-value	Enrichment (N, B, n, b)*	Genes
					GRIN1 - glutamate receptor, ionotropic, n-methyl d-aspartate 1
					GRIN2B - glutamate receptor, ionotropic, n-methyl d-aspartate 2b
					DRD5 - dopamine receptor d5
					RNF180 - ring finger protein 180
					CRHR1 - corticotropin releasing hormone receptor 1
					ADCY8 - adenylate cyclase 8 (brain)
					ARC - activity-regulated cytoskeleton-associated protein
					NR2E1 - nuclear receptor subfamily 2, group e, member 1
					CACNA1C - calcium channel, voltage-dependent, l type, alpha 1c subunit
					CUX2 - cut-like homeobox 2
					FOXA2 - forkhead box a2
GO:0044700	single organism signaling	8.04E-05	2.09E-01	2.62 (18004,750,183,20)	
					NTF4 - neurotrophin 4
					CACNG2 - calcium channel, voltage-dependent, gamma subunit 2
					GNG7 - guanine nucleotide binding protein (g protein), gamma 7
					GRIN1 - glutamate receptor, ionotropic, n-methyl d-aspartate 1
					KCNA1 - potassium voltage-gated channel, shaker-related subfamily, member 1 (episodic ataxia with myokymia)
					GRIN2B - glutamate receptor, ionotropic, n-methyl d-aspartate 2b
					GABBR1 - gamma-aminobutyric acid (gaba) b receptor, 1
					DRD5 - dopamine receptor d5
					CPLX1 - complexin 1
					GJA3 - gap junction protein, alpha 3, 46kda
					KCNJ9 - potassium inwardly-rectifying channel, subfamily j, member 9
					SYT5 - synaptotagmin v
					GABRG3 - gamma-aminobutyric acid (gaba) a receptor, gamma 3
					KCNV1 - potassium channel, subfamily v, member 1
					PANX2 - pannexin 2
					PTHLH - parathyroid hormone-like hormone
					ADCY8 - adenylate cyclase 8 (brain)
					PCDHB6 - protocadherin beta 6
					CACNA1C - calcium channel, voltage-dependent, l type, alpha 1c subunit
					IHH - indian hedgehog
GO:0023052	signaling	8.19E-05	1.78E-01	2.62 (18004,751,183,20)	
					NTF4 - neurotrophin 4
					CACNG2 - calcium channel, voltage-dependent, gamma subunit 2
					GNG7 - guanine nucleotide binding protein (g protein), gamma 7
					GRIN1 - glutamate receptor, ionotropic, n-methyl d-aspartate 1
					KCNA1 - potassium voltage-gated channel, shaker-related subfamily, member 1 (episodic ataxia with myokymia)
					GRIN2B - glutamate receptor, ionotropic, n-methyl d-aspartate 2b
					GABBR1 - gamma-aminobutyric acid (gaba) b receptor, 1
					DRD5 - dopamine receptor d5
					CPLX1 - complexin 1
					GJA3 - gap junction protein, alpha 3, 46kda
					KCNJ9 - potassium inwardly-rectifying channel, subfamily j, member 9
					SYT5 - synaptotagmin v
					GABRG3 - gamma-aminobutyric acid (gaba) a receptor, gamma 3
					KCNV1 - potassium channel, subfamily v, member 1
					PANX2 - pannexin 2
					PTHLH - parathyroid hormone-like hormone
					ADCY8 - adenylate cyclase 8 (brain)
					PCDHB6 - protocadherin beta 6
					CACNA1C - calcium channel, voltage-dependent, l type, alpha 1c subunit

GO term	Description	P value	FDR q-value	Enrichment (N, B, n, b)*	Genes
					IHH - indian hedgehog
GO:0051960	regulation of nervous system development	8.26E-05	1.54E-01	2.91 (18004,575,183,17)	
					GRIN1 - glutamate receptor, ionotropic, n-methyl d-aspartate 1
					NTF4 - neurotrophin 4
					PAX6 - paired box 6
					SARM1 - sterile alpha and tir motif containing 1
					HES3 - hairy and enhancer of split 3 (drosophila)
					CFL1 - cofilin 1 (non-muscle)
					WNT3A - wingless-type mmtv integration site family, member 3a
					THBS2 - thrombospondin 2
					OPCML - opioid binding protein/cell adhesion molecule-like
					TENM4 - teneurin transmembrane protein 4
					CBLN2 - cerebellin 2 precursor
					NR2E1 - nuclear receptor subfamily 2, group e, member 1
					DCC - deleted in colorectal carcinoma
					NEUROG1 - neurogenin 1
					CUX2 - cut-like homeobox 2
					STMN2 - stathmin-like 2
					FOXA2 - forkhead box a2
GO:2000543	positive regulation of gastrulation	8.30E-05	1.35E-01	32.79 (18004,9,183,3)	
					WNT3A - wingless-type mmtv integration site family, member 3a
					TENM4 - teneurin transmembrane protein 4
					FOXA2 - forkhead box a2
GO:0007154	cell communication	1.11E-04	1.61E-01	2.42 (18004,893,183,22)	
					NTF4 - neurotrophin 4
					GNG7 - guanine nucleotide binding protein (g protein), gamma 7
					KREMEN2 - kringle containing transmembrane protein 2
					GRIN1 - glutamate receptor, ionotropic, n-methyl d-aspartate 1
					CACNG2 - calcium channel, voltage-dependent, gamma subunit 2
					KCNA1 - potassium voltage-gated channel, shaker-related subfamily, member 1 (episodic ataxia with myokymia)
					GRIN2B - glutamate receptor, ionotropic, n-methyl d-aspartate 2b
					GABBR1 - gamma-aminobutyric acid (gaba) b receptor, 1
					DRD5 - dopamine receptor d5
					CPLX1 - complexin 1
					GJA3 - gap junction protein, alpha 3, 46kda
					KCNJ9 - potassium inwardly-rectifying channel, subfamily j, member 9
					SYT5 - synaptotagmin v
					GABRG3 - gamma-aminobutyric acid (gaba) a receptor, gamma 3
					PCSK9 - proprotein convertase subtilisin/kexin type 9
					PANX2 - pannexin 2
					KCNV1 - potassium channel, subfamily v, member 1
					ADCY8 - adenylate cyclase 8 (brain)
					PTHLH - parathyroid hormone-like hormone
					PCDHB6 - protocadherin beta 6
					CACNA1C - calcium channel, voltage-dependent, l type, alpha 1c subunit
					IHH - indian hedgehog
GO:2000047	regulation of cell-cell adhesion mediated by cadherin	1.18E-04	1.53E-01	29.51 (18004,10,183,3)	
					RGCC - regulator of cell cycle
					WNT3A - wingless-type mmtv integration site family, member 3a
					FOXA2 - forkhead box a2
GO:0042596	fear response	1.51E-04	1.79E-01	14.58 (18004,27,183,4)	

GO term	Description	P value	FDR q-value	Enrichment (N, B, n, b)*	Genes
					CRHR1 - corticotropin releasing hormone receptor 1
					GNG7 - guanine nucleotide binding protein (g protein), gamma 7
					GRIN2B - glutamate receptor, ionotropic, n-methyl d-aspartate 2b
					NR2E1 - nuclear receptor subfamily 2, group e, member 1
GO:0030814	regulation of cAMP metabolic process	1.56E-04	1.69E-01	6.09 (18004,113,183,7)	
					GRM8 - glutamate receptor, metabotropic 8
					GNG7 - guanine nucleotide binding protein (g protein), gamma 7
					CRHR1 - corticotropin releasing hormone receptor 1
					GABBR1 - gamma-aminobutyric acid (gaba) b receptor, 1
					DRD5 - dopamine receptor d5
					PTHLH - parathyroid hormone-like hormone
					MC3R - melanocortin 3 receptor
GO:0048505	regulation of timing of cell differentiation	1.61E-04	1.61E-01	26.83 (18004,11,183,3)	
					PAX6 - paired box 6
					NR2E1 - nuclear receptor subfamily 2, group e, member 1
					HES3 - hairy and enhancer of split 3 (drosophila)
GO:0007611	learning or memory	1.62E-04	1.51E-01	4.59 (18004,193,183,9)	
					GRIN1 - glutamate receptor, ionotropic, n-methyl d-aspartate 1
					NTF4 - neurotrophin 4
					CRHR1 - corticotropin releasing hormone receptor 1
					GRIN2B - glutamate receptor, ionotropic, n-methyl d-aspartate 2b
					DRD5 - dopamine receptor d5
					ARC - activity-regulated cytoskeleton-associated protein
					ADCY8 - adenylate cyclase 8 (brain)
					CUX2 - cut-like homeobox 2
					CACNA1C - calcium channel, voltage-dependent, l type, alpha 1c subunit
GO:0030802	regulation of cyclic nucleotide biosynthetic process	1.83E-04	1.59E-01	5.94 (18004,116,183,7)	
					GRM8 - glutamate receptor, metabotropic 8
					GNG7 - guanine nucleotide binding protein (g protein), gamma 7
					CRHR1 - corticotropin releasing hormone receptor 1
					GABBR1 - gamma-aminobutyric acid (gaba) b receptor, 1
					DRD5 - dopamine receptor d5
					PTHLH - parathyroid hormone-like hormone
					MC3R - melanocortin 3 receptor
GO:0040034	regulation of development, heterochronic	2.12E-04	1.73E-01	24.60 (18004,12,183,3)	
					PAX6 - paired box 6
					NR2E1 - nuclear receptor subfamily 2, group e, member 1
					HES3 - hairy and enhancer of split 3 (drosophila)
GO:1900371	regulation of purine nucleotide biosynthetic process	2.15E-04	1.65E-01	5.79 (18004,119,183,7)	
					GRM8 - glutamate receptor, metabotropic 8
					GNG7 - guanine nucleotide binding protein (g protein), gamma 7
					CRHR1 - corticotropin releasing hormone receptor 1
					GABBR1 - gamma-aminobutyric acid (gaba) b receptor, 1
					DRD5 - dopamine receptor d5
					PTHLH - parathyroid hormone-like hormone
					MC3R - melanocortin 3 receptor
GO:0030808	regulation of nucleotide biosynthetic process	2.15E-04	1.55E-01	5.79 (18004,119,183,7)	
					GRM8 - glutamate receptor, metabotropic 8
					GNG7 - guanine nucleotide binding protein (g protein), gamma 7
					CRHR1 - corticotropin releasing hormone receptor 1
					GABBR1 - gamma-aminobutyric acid (gaba) b receptor, 1
					DRD5 - dopamine receptor d5

GO term	Description	P value	FDR q-value	Enrichment (N, B, n, b)*	Genes
					PTHLH - parathyroid hormone-like hormone
					MC3R - melanocortin 3 receptor
GO:0050767	regulation of neurogenesis	2.62E-04	1.80E-01	2.86 (18004,516,183,15)	
					GRIN1 - glutamate receptor, ionotropic, n-methyl d-aspartate 1
					NTF4 - neurotrophin 4
					PAX6 - paired box 6
					SARM1 - sterile alpha and tir motif containing 1
					HES3 - hairy and enhancer of split 3 (drosophila)
					CFL1 - cofilin 1 (non-muscle)
					WNT3A - wingless-type mmtv integration site family, member 3a
					OPCML - opioid binding protein/cell adhesion molecule-like
					TENM4 - teneurin transmembrane protein 4
					NR2E1 - nuclear receptor subfamily 2, group e, member 1
					DCC - deleted in colorectal carcinoma
					NEUROG1 - neurogenin 1
					CUX2 - cut-like homeobox 2
					STMN2 - stathmin-like 2
					FOXA2 - forkhead box a2
GO:0010470	regulation of gastrulation	2.62E-04	1.71E-01	12.69 (18004,31,183,4)	
					WNT3A - wingless-type mmtv integration site family, member 3a
					DKK1 - dickkopf wnt signaling pathway inhibitor 1
					TENM4 - teneurin transmembrane protein 4
					FOXA2 - forkhead box a2
GO:0010455	positive regulation of cell fate commitment	2.74E-04	1.70E-01	22.70 (18004,13,183,3)	
					WNT3A - wingless-type mmtv integration site family, member 3a
					DKK1 - dickkopf wnt signaling pathway inhibitor 1
					PAX6 - paired box 6
GO:0050773	regulation of dendrite development	2.76E-04	1.63E-01	6.71 (18004,88,183,6)	
					GRIN1 - glutamate receptor, ionotropic, n-methyl d-aspartate 1
					DCC - deleted in colorectal carcinoma
					NR2E1 - nuclear receptor subfamily 2, group e, member 1
					SARM1 - sterile alpha and tir motif containing 1
					CUX2 - cut-like homeobox 2
					CFL1 - cofilin 1 (non-muscle)
GO:0048814	regulation of dendrite morphogenesis	2.80E-04	1.59E-01	8.63 (18004,57,183,5)	
					GRIN1 - glutamate receptor, ionotropic, n-methyl d-aspartate 1
					NR2E1 - nuclear receptor subfamily 2, group e, member 1
					SARM1 - sterile alpha and tir motif containing 1
					CUX2 - cut-like homeobox 2
					CFL1 - cofilin 1 (non-muscle)
GO:0045664	regulation of neuron differentiation	2.91E-04	1.58E-01	3.13 (18004,409,183,13)	
					GRIN1 - glutamate receptor, ionotropic, n-methyl d-aspartate 1
					NTF4 - neurotrophin 4
					PAX6 - paired box 6
					SARM1 - sterile alpha and tir motif containing 1
					HES3 - hairy and enhancer of split 3 (drosophila)
					CFL1 - cofilin 1 (non-muscle)
					WNT3A - wingless-type mmtv integration site family, member 3a
					DCC - deleted in colorectal carcinoma
					NR2E1 - nuclear receptor subfamily 2, group e, member 1
					CUX2 - cut-like homeobox 2
					NEUROG1 - neurogenin 1

GO term	Description	P value	FDR q-value	Enrichment (N, B, n, b)*	Genes
					STMN2 - stathmin-like 2
					FOXA2 - forkhead box a2
GO:0007613	memory	3.31E-04	1.73E-01	6.49 (18004,91,183,6)	
					GRIN1 - glutamate receptor, ionotropic, n-methyl d-aspartate 1
					NTF4 - neurotrophin 4
					CRHR1 - corticotropin releasing hormone receptor 1
					GRIN2B - glutamate receptor, ionotropic, n-methyl d-aspartate 2b
					ADCY8 - adenylate cyclase 8 (brain)
					CUX2 - cut-like homeobox 2
GO:0050890	cognition	4.57E-04	2.29E-01	3.99 (18004,222,183,9)	
					GRIN1 - glutamate receptor, ionotropic, n-methyl d-aspartate 1
					NTF4 - neurotrophin 4
					CRHR1 - corticotropin releasing hormone receptor 1
					GRIN2B - glutamate receptor, ionotropic, n-methyl d-aspartate 2b
					DRD5 - dopamine receptor d5
					ARC - activity-regulated cytoskeleton-associated protein
					ADCY8 - adenylate cyclase 8 (brain)
					CUX2 - cut-like homeobox 2
					CACNA1C - calcium channel, voltage-dependent, l type, alpha 1c subunit
GO:0030799	regulation of cyclic nucleotide metabolic process	4.64E-04	2.24E-01	5.10 (18004,135,183,7)	
					GRM8 - glutamate receptor, metabotropic 8
					GNG7 - guanine nucleotide binding protein (g protein), gamma 7
					CRHR1 - corticotropin releasing hormone receptor 1
					GABBR1 - gamma-aminobutyric acid (gaba) b receptor, 1
					DRD5 - dopamine receptor d5
					PTHLH - parathyroid hormone-like hormone
					MC3R - melanocortin 3 receptor
GO:2000648	positive regulation of stem cell proliferation	4.82E-04	2.24E-01	7.69 (18004,64,183,5)	
					TBX3 - t-box 3
					PAX6 - paired box 6
					NR2E1 - nuclear receptor subfamily 2, group e, member 1
					IHH - indian hedgehog
					CHRD - chordin
GO:0042660	positive regulation of cell fate specification	6.08E-04	2.73E-01	49.19 (18004,4,183,2)	
					WNT3A - wingless-type mmtv integration site family, member 3a
					PAX6 - paired box 6
GO:0090381	regulation of heart induction	6.08E-04	2.64E-01	49.19 (18004,4,183,2)	
					WNT3A - wingless-type mmtv integration site family, member 3a
					DKK1 - dickkopf wnt signaling pathway inhibitor 1
GO:0051963	regulation of synapse assembly	6.43E-04	2.70E-01	10.09 (18004,39,183,4)	
					GRIN1 - glutamate receptor, ionotropic, n-methyl d-aspartate 1
					THBS2 - thrombospondin 2
					CBLN2 - cerebellin 2 precursor
					CUX2 - cut-like homeobox 2
GO:0048598	embryonic morphogenesis	6.93E-04	2.82E-01	3.01 (18004,392,183,12)	
					TBX3 - t-box 3
					WNT3A - wingless-type mmtv integration site family, member 3a
					FZD5 - frizzled family receptor 5
					DKK1 - dickkopf wnt signaling pathway inhibitor 1
					PAX6 - paired box 6
					TENM4 - teneurin transmembrane protein 4

GO term	Description	P value	FDR q-value	Enrichment (N, B, n, b)*	Genes
					ALX3 - alx homeobox 3
					NEUROG1 - neurogenin 1
					HES3 - hairy and enhancer of split 3 (drosophila)
					IHH - indian hedgehog
					CFL1 - cofilin 1 (non-muscle)
					CHRD - chordin
GO:0055085	transmembrane transport	7.07E-04	2.79E-01	2.08 (18004,1090,183,23)	
					GRIN1 - glutamate receptor, ionotropic, n-methyl d-aspartate 1
					CACNG2 - calcium channel, voltage-dependent, gamma subunit 2
					GRIN2B - glutamate receptor, ionotropic, n-methyl d-aspartate 2b
					KCNA1 - potassium voltage-gated channel, shaker-related subfamily, member 1 (episodic ataxia with myokymia)
					SLC39A3 - solute carrier family 39 (zinc transporter), member 3
					OCA2 - oculocutaneous albinism ii
					SLC16A12 - solute carrier family 16, member 12
					RASA3 - ras p21 protein activator 3
					NDUFA13 - nadh dehydrogenase (ubiquinone) 1 alpha subcomplex, 13
					TTYH3 - tweety homolog 3 (drosophila)
					GJA3 - gap junction protein, alpha 3, 46kda
					KCNJ9 - potassium inwardly-rectifying channel, subfamily j, member 9
					GABRG3 - gamma-aminobutyric acid (gaba) a receptor, gamma 3
					ATP5D - atp synthase, h+ transporting, mitochondrial f1 complex, delta subunit
					ZACN - zinc activated ligand-gated ion channel
					ATP8B3 - atpase, aminophospholipid transporter, class i, type 8b, member 3
					PANX2 - pannexin 2
					KCNV1 - potassium channel, subfamily v, member 1
					ADCY8 - adenylate cyclase 8 (brain)
					SLC39A7 - solute carrier family 39 (zinc transporter), member 7
					CACNA1C - calcium channel, voltage-dependent, l type, alpha 1c subunit
					TPTE - transmembrane phosphatase with tensin homology
					SLCO2B1 - solute carrier organic anion transporter family, member 2b1
GO:0042659	regulation of cell fate specification	7.53E-04	2.89E-01	16.40 (18004,18,183,3)	
					WNT3A - wingless-type mmtv integration site family, member 3a
					DKK1 - dickkopf wnt signaling pathway inhibitor 1
					PAX6 - paired box 6

Enrichment (N, B, n, b) is defined as follows: N : Total number of genes; B: Total number of genes associated with a specific GO term; n: Number of genes in the top of the user's input list or in the target set when appropriate; b: Number of genes in the intersection; Enrichment = (b/n) / (B/N).

Supplementary Table 5. Associations (false discovery rate corrected P value < 0.1) between gene expression in white blood cells and methylation in a cohort of older adults (Rotterdam Study) for CpGs significantly associated with maternal plasma folate in newborns in the meta-analysis

Probe ID	Expressed Gene	CpG	Coef	SE	P	FDR
ILMN_1774256	*PRSS21*	cg01232511	-2.53	0.22	7.86E-28	2.05E-24
ILMN_1774256	*PRSS21*	cg02296564	-3.89	0.35	1.38E-26	1.80E-23
ILMN_1774256	*PRSS21*	cg05635274	-5.06	0.47	4.06E-25	3.52E-22
ILMN_1774256	*PRSS21*	cg22730830	-2.74	0.26	6.30E-25	4.10E-22
ILMN_1786426	*IGFLR1*	cg24860092	-10.86	1.05	1.30E-23	6.79E-21
ILMN_2339796	*CDC16*	cg04718414	-10.86	1.30	3.22E-16	1.40E-13
ILMN_1727389	*CDC16*	cg04718414	-10.09	1.25	2.66E-15	9.88E-13
ILMN_1692072	*CENPBD1*	cg08539210	-3.48	0.44	8.05E-15	2.39E-12
ILMN_1692072	*CENPBD1*	cg13969584	-3.62	0.46	8.25E-15	2.39E-12
ILMN_2359945	*CES1*	cg03880033	-3.88	0.55	3.05E-12	7.95E-10
ILMN_2256765	*CDC16*	cg04718414	-8.29	1.20	1.23E-11	2.92E-09
ILMN_1655077	*PRDM1*	cg00614392	4.94	0.80	9.53E-10	2.07E-07
ILMN_2294784	*PRDM1*	cg00614392	3.39	0.57	4.46E-09	8.94E-07
ILMN_1778010	*IL32*	cg05635274	-4.73	0.82	1.21E-08	2.25E-06
ILMN_1675386	*CES1;CES1P1*	cg03880033	-1.44	0.31	2.74E-06	4.75E-04
ILMN_2368530	*IL32*	cg05635274	-5.36	1.20	8.60E-06	0.0014
ILMN_2154671	*COX6B1*	cg24860092	-2.31	0.52	1.03E-05	0.0016
ILMN_1770765	*PRSS30P*	cg05635274	-3.22	0.76	2.64E-05	0.0038
ILMN_1796417	*ASNS*	cg26284540	2.98	0.71	2.94E-05	0.0040
ILMN_1734895	*SFT2D1*	cg01243879	1.96	0.47	3.57E-05	0.0046
ILMN_1667893	*TNS3*	cg20258698	3.02	0.74	5.37E-05	0.0067
ILMN_1804148	*TMED4*	cg04674755	-2.53	0.63	6.02E-05	0.0071
ILMN_1655167	*ZNF502*	cg14768256	-0.96	0.24	7.48E-05	0.0085
ILMN_1663080	*LFNG*	cg12400790	-5.14	1.31	9.65E-05	0.0105
ILMN_1767509	*DEF8*	cg08539210	-2.53	0.65	1.13E-04	0.0118
ILMN_1735058	*CHST12*	cg12400790	4.82	1.29	1.94E-04	0.0195
ILMN_1786426	*IGFLR1*	cg15474831	-9.83	2.66	2.37E-04	0.0228
ILMN_1794364	*CTSW*	cg02230254	-3.97	1.10	3.09E-04	0.0287
ILMN_2339006	*KIAA0564*	cg14496523	2.59	0.72	3.65E-04	0.0328
ILMN_1705617	*CFL1*	cg15777472	1.47	0.42	4.49E-04	0.0390
ILMN_1778010	*IL32*	cg05185364	-5.60	1.61	5.46E-04	0.0458
ILMN_2374352	*DBNDD1*	cg08539210	-2.61	0.76	6.57E-04	0.0535
ILMN_2188204	*ATG12*	cg06682875	1.40	0.41	7.01E-04	0.0538
ILMN_1723021	*ICMT*	cg19694465	1.70	0.50	7.03E-04	0.0538
ILMN_1662741	*LPAR2*	cg03233793	2.68	0.80	8.79E-04	0.0642
ILMN_1697561	*FBXL16*	cg09852871	-2.38	0.71	9.01E-04	0.0642
ILMN_1732023	*PRDM7*	cg13969584	1.33	0.40	9.12E-04	0.0642
ILMN_1728535	*COPZ1*	cg20474425	1.67	0.51	1.04E-03	0.0715
ILMN_2268156	*LFNG*	cg04021544	0.66	0.20	1.09E-03	0.0725
ILMN_1793017	*DGKQ*	cg05448404	-1.68	0.51	1.11E-03	0.0725
ILMN_1767509	*DEF8*	cg13969584	-2.20	0.68	1.28E-03	0.0815
ILMN_1724437	*GCAT*	cg07059402	-1.47	0.46	1.39E-03	0.0865
ILMN_1732023	*PRDM7*	cg08539210	1.22	0.38	1.58E-03	0.0954

Probe ID: probe ID from the Illumina HumanHT12v4 Expression Beadchip; Expressed Gene: Gene corresponding to the probe ID; Coef: the model coefficient representing the difference in gene expression per 1% increase in methylation of the specified CpG-site; SE: standard error; P: Uncorrected P value; FDR: False discovery rate corrected P value. Only statistically significant associations after FDR-correction (corrected P < 0.1) are presented. The mRNA gene expression data was quantile-normalized to the median distribution and subsequently log2-transformed. The probe and sample means were centered to zero. Models were adjusted for white blood cell proportions, age, sex and batch effects.

Supplementary Methods

MoBa

MoBa study population

Participants in the current analysis include mother-offspring pairs from a substudy of the Norwegian Mother and Child Cohort Study (MoBa)[1,2] designed to evaluate the association between maternal plasma folate during pregnancy and childhood asthma status at 3 years of age.[3] We previously reported an association between maternal smoking during pregnancy and differential DNA methylation in 1,062 of the Moba newborns.[4] We subsequently measured DNA methylation in an additional 685 newborns (including 221 with maternal plasma folate measurements) and following separate quality control, combined the two datasets as described below. We refer to these two datasets here as MoBa1 and MoBa2. The current analysis includes the 1,283 children who had cord blood DNA methylation measurements and maternal plasma folate measured during pregnancy at approximately 18 weeks gestation (N=1,062 from MoBa1, N=221 from MoBa2). The year of birth for participants in our analysis ranged from 2002 to 2004. The study was approved by the Regional Committee for Ethics in Medical Research, the Norwegian Data Inspectorate and the Institutional Review Board of the National Institute of Environmental Health Sciences, USA, and written informed consent was provided by all mothers participating.

MoBa maternal folate, vitamin B12, homocysteine, vitamin D, and SNP measurements

Maternal blood samples were drawn during pregnancy (median weeks gestation=18 weeks, 25th-75th percentile=16-21 weeks) in EDTA lined tubes, centrifuged within 30 minutes after collection, and stored at 4°C in the hospital where they were collected. Samples were then shipped overnight to the Biobank of MoBa at the Norwegian Institute of Public Health in Oslo. Upon receipt (1-2 days after blood collection), plasma was aliquoted onto polypropylene microtiter plates, sealed with heat-sealing foil sheets, and stored at -80°C. Plasma folate concentration was measured at Bevital AS (www.bevital.no) by microbiological assay, using a chloramphenicol-resistant strain of *Lactobacillus casei*,[5] which measures biologically active folate species, predominantly 5-methyl-tetrahydrofolate. The CV for this assay corresponds to 4% within-day and 5% between days, at population median.

Plasma cobalamin (vitamin B12) was determined by a *Lactobacillus leichmannii* microbiological assay.[6] Plasma levels of total homocysteine (tHcy), a marker of folate and cobalamin status, were assayed using a gas chromatography-mass spectrometry method based on methylchloroformate derivatization.[7] Plasma 25-hydroxyvitamin D2 and D3 was measured by liquid chromatography/tandem mass spectrometry[8] and the sum of 25-hydroxyvitamin D2 and D3 was used as a measure for vitamin D status.[9] The lower limit of detection and CV for each of these biomarkers is published elsewhere (www.bevital.no).

Two MTHFR SNPs were analyzed. These SNPs were genotyped using high-level multiplex genotyping based on matrix-assisted laser desorption/ionization time-of-flight mass spectrometry (MALDI-TOF MS) in the LOCUS laboratory as previously described.[10]

MoBa covariates

Information on maternal age, parity, and maternal education was collected via questionnaires completed by the mother or from birth registry records. Parity was categorized as 0, 1, 2, or >=3 births. Maternal educational level was categorized into less than high school/secondary school, high school/secondary school completion, some college or university, or 4 years of college/university or more. Maternal smoking during pregnancy (yes vs. no) was assessed by maternal questionnaire and plasma cotinine, a biomarker of tobacco smoking, measured by liquid chromatography - tandem mass spectrometry[11] at approximately 18 weeks gestation. If a mother reported not smoking but had plasma cotinine ≥ 56.8 nmol/L she was categorized as a smoker. If a mother reported that she stopped in pregnancy and had cotinine values < 56.8 nmol/L she was classified as a nonsmoker. Maternal age was included as a continuous variable.

MoBa methylation measurements

For the DNA methylation measurements and quality control for the MoBa1[4] and MoBa2 participants, umbilical cord blood samples were collected and frozen at birth at -80° Celsius. All biological material was obtained from the Biobank of the MoBa study.[2] Bisulfite conversion was performed using the EZ-96 DNA Methylation kit (Zymo Research Corporation, Irvine, CA) and DNA methylation was measured at 485,577 CpGs in cord blood using Illumina's Infinium HumanMethylation450 BeadChip.[12] Raw intensity (.idat) files were handled in R using the *minfi* package[13] to calculate the methylation level at each CpG as the beta-value (β=intensity of the methylated allele (M)/(intensity of the unmethylated allele (U) + intensity of the methylated allele (M) + 100)) and the data was exported for quality control and processing.

Probe and sample-specific quality control was performed in MoBa 1 and 2 datasets separately. Control probes (N=65) and probes on X (N=11,230) and Y (N=416) chromosomes were excluded in both datasets. Remaining CpGs missing >10% of methylation data were also removed (N=20 in MoBa1, none in MoBa2). Samples indicated by Illumina to have failed or have an average detection P value across all probes < 0.05 (N=49 MoBa1, N=35 MoBa2) and samples with gender mismatch (N=13 MoBa1, N=8 MoBa2) were also removed.

For each dataset, we accounted for the two different probe designs by applying the intra-array normalization strategy Beta Mixture Quantile dilation (BMIQ).[14] The Empirical Bayes method via *ComBat* was applied for batch correction using the *sva* package in *R.*[15] The two datasets were then combined and *ComBat* was run again to account for the potential batch effect of measuring methylation at two different time periods. The final MoBa dataset for this analysis included N=1,283 participants with measured DNA methylation, maternal plasma folate, and covariates and restricted to CpGs common to both MoBa1 and MoBa2 datasets (N=473,731).

Generation R

Generation R study population

The Generation R Study is a population-based prospective cohort study from fetal life onwards in Rotterdam, the Netherlands.[16] Assessments in pregnant women consisted of physical examinations, fetal ultrasounds, biological samples, and questionnaires.[17] All children were born between April 2002 and January 2006. The study has been approved by the Medical Ethical Committee of the Erasmus University Medical Centre and written consent was obtained from participating parents of their children. For the current study, data was available for 790 Caucasian mothers and their children with information on plasma folate and DNA-methylation; 713 with additional complete data on covariates.

Generation R maternal folate, vitamin B12, homocysteine, vitamin D, and SNP measurements

Venous blood samples were drawn at enrollment of the mothers in early pregnancy (median: 12.9 weeks gestation; 25th-75th percentile=12.1-13.9 weeks) and stored at room temperature for a maximum of 3 hours. At birth, cord blood samples were collected by midwifes or obstetricians [17]. Samples were transported to a laboratory facility of the regional laboratory in Rotterdam, Netherlands (Star-Medisch Diagnostisch Centrum) for additional processing and storage at −80°C. The samples were analyzed at the Department of Clinical Chemistry at the Erasmus University Medical Centre, Rotterdam. After thawing, the folate, vitamin B12, and homocysteine levels were analysed using an immunoelectrochemoluminescence assay on the Architect System (Abbott Diagnostics B.V., Hoofddorp, the Netherlands).[18] The between-run coefficient of variation for plasma folate was 8.9% at 5.6 nmol/l, 2.5% at 16.6 nmol/l and 1.5% at 33.6 nmol/l, with an analytical range of 1.8 - 45.3 nmol/l. The between-run coefficient of variation for serum vitamin B12 was 3.6% at 142 pmol/l, 7.5% at 308 pmol/l and 3.1% at 633 pmol/l, with an analytical range of 44 - 1476 pmol/l.[18] The between-run coefficient of variation for plasma homocysteine was 3.1% at 7.2 µmol/l, 3.1% at 12.9 µmol/l and 2.1% at 26.1 µmol/l, with an analytic range of 1 - 50 µmol/l. Folate, vitamin B12, and homocysteine levels were available in 80.8%, 79.9% and 76.5% of the mothers, respectively, and folate was available in 71.2% of the children.

We collected maternal venous blood samples in mid gestation [median 20.5 (range 18.1–24.9) weeks of gestation] to assess vitamin D levels. Levels of 25-hydroxyvitamin D were analysed from stored frozen samples at -80°C.[17] Measurements of maternal 25-hydroxyvitamin D levels were conducted at the Eyles Laboratory at the Queensland Brain Institute, University of Queensland, Australia. Total 25-hydroxyvitamin D level was reported as the sum of 25-hydroxyvitamin D2 and D3 species measured in plasma using a modification of a method previously described.[19,20] Inter-assay inaccuracy and imprecision were assessed at four concentration levels for 25-hydroxyvitamin D3 (48.3, 49.4, 76.4, and 139.2 nmol/L) and a single level (32.3 nmol/L) for 25-hydroxyvitamin D2 using certified reference materials purchased from the National Institute of Standards and Technology (NIST) (NIST SRM 972a levels 1–4) and were excellent at all concentration levels tested. Interassay inaccuracy and imprecision were both < 10% for 25-hydroxyvitamin D3 and < 17% for 25-

hydroxyvitamin D2. Assay repeatability was assessed via replicate analysis of an independent reference material (NIST SRM1950, 61.9 nmol/L 25-hydroxyvitamin D3), with interassay inaccuracy and imprecision both < 11% (n = 343).

Mothers were genotyped for rs1801131 and rs1801133 using a TaqMan allelic discrimination assay (Applied Biosystems) and Abgene QPCR ROX mix. The genotyping reaction was amplified using the GeneAmp PCR system 9600 [95°C (15 min), then 40 cycles of 94°C (15 s) and 60°C (1 min)]. Fluorescence was detected on the 7900HT Fast Real-Time PCR System (Applied Biosystems) and individual genotypes were determined using SDS software (version 2.3, Applied Biosystems).

Generation R covariates

Information on maternal age, parity, and maternal education was collected by questionnaires at enrollment. Parity was categorized as nulliparity or multiparity, and maternal educational level as lower (none, primary or secondary education) or higher (more than secondary education) education. Maternal smoking during pregnancy was assessed by a questionnaire at enrollment. Information about maternal smoking during other periods of pregnancy was obtained by postal questionnaires sent in the second and third trimesters of pregnancy. Response rates for these questionnaires were 91%, 80%, 77%, respectively. Based on all three questionnaires, maternal smoking was classified into any reported active smoking during pregnancy (no, yes).[17]

Generation R methylation measurements

DNA was extracted from cord blood samples of 979 Caucasian children. Using the EZ-96 DNA Methylation kit (Shallow-well) (Zymo Research Corporation, Irvine, USA), 500 ng DNA per sample underwent bisulfite conversion. Samples were transferred onto 96-well plates in a random order. Samples were processed with Illumina's Infinium HumanMethylation450 BeadChip (Illumina Inc., San Diego, USA). Quality control of analyzed samples was performed using standardized criteria. Samples were excluded due to sample call rate <99% (n=7) or poor bisulfite conversion (n=1). In addition, 2 samples were excluded because of a gender mismatch and 1 sample because of a retracted informed consent, leaving a total of 969 samples in the statistical analysis.

Probes with a single nucleotide polymorphism in the single base extension site with a frequency of > 1% in the GoNLv4 reference panel were excluded, as were probes with non-optimal binding (non-mapping or mapping multiple times to either the normal or the bisulphite-converted genome), resulting in the exclusion of 49,564 probes, leaving a total of 436,013 probes in the analysis.

Data were normalized with DASES normalization using a pipeline adapted from that developed by Touleimat and Tost.[21] DASES normalization includes background adjustment, between-array normalization applied to type I and type II probes separately, and dye bias correction applied to type I and type II probes separately. DASES is based on the DASEN method, but adds the dye bias correction, which is not included in DASEN.[22] Beta-values were calculated for all CpG sites.

Supplementary References

1. Magnus, P. *et al.* Cohort profile: the Norwegian Mother and Child Cohort Study (MoBa). *Int J Epidemiol* **35**, 1146-50 (2006).
2. Ronningen, K.S. *et al.* The biobank of the Norwegian Mother and Child Cohort Study: a resource for the next 100 years. *Eur J Epidemiol* **21**, 619-25 (2006).
3. Haberg, S.E. *et al.* Maternal folate levels in pregnancy and asthma in children at age 3 years. *J Allergy Clin Immunol* **127**, 262-4, 264 e1 (2011).
4. Joubert, B.R. *et al.* 450K epigenome-wide scan identifies differential DNA methylation in newborns related to maternal smoking during pregnancy. *Environ Health Perspect* **120**, 1425-31 (2012).
5. O'Broin, S. & Kelleher, B. Microbiological assay on microtitre plates of folate in serum and red cells. *J Clin Pathol* **45**, 344-7 (1992).
6. Kelleher, B.P. & Broin, S.D. Microbiological assay for vitamin B12 performed in 96-well microtitre plates. *J Clin Pathol* **44**, 592-5 (1991).
7. Windelberg, A., Arseth, O., Kvalheim, G. & Ueland, P.M. Automated assay for the determination of methylmalonic acid, total homocysteine, and related amino acids in human serum or plasma by means of methylchloroformate derivatization and gas chromatography-mass spectrometry. *Clin Chem* **51**, 2103-9 (2005).
8. Midttun, O. & Ueland, P.M. Determination of vitamins A, D and E in a small volume of human plasma by a high-throughput method based on liquid chromatography/tandem mass spectrometry. *Rapid Commun Mass Spectrom* **25**, 1942-8 (2011).
9. Magnus, M.C. *et al.* Prospective study of maternal mid-pregnancy 25-hydroxyvitamin D level and early childhood respiratory disorders. *Paediatr Perinat Epidemiol* **27**, 532-41 (2013).
10. Meyer, K., Fredriksen, A. & Ueland, P.M. High-level multiplex genotyping of polymorphisms involved in folate or homocysteine metabolism by matrix-assisted laser desorption/ionization mass spectrometry. *Clin Chem* **50**, 391-402 (2004).
11. Midttun, O., Hustad, S. & Ueland, P.M. Quantitative profiling of biomarkers related to B-vitamin status, tryptophan metabolism and inflammation in human plasma by liquid chromatography/tandem mass spectrometry. *Rapid Commun Mass Spectrom* **23**, 1371-9 (2009).
12. Bibikova, M. *et al.* High density DNA methylation array with single CpG site resolution. *Genomics* **98**, 288-95 (2011).
13. Aryee, M.J. *et al.* Minfi: a flexible and comprehensive Bioconductor package for the analysis of Infinium DNA methylation microarrays. *Bioinformatics* **30**, 1363-9 (2014).
14. Teschendorff, A.E. *et al.* A beta-mixture quantile normalization method for correcting probe design bias in Illumina Infinium 450 k DNA methylation data. *Bioinformatics* **29**, 189-96 (2013).
15. Johnson, W.E., Li, C. & Rabinovic, A. Adjusting batch effects in microarray expression data using empirical Bayes methods. *Biostatistics* **8**, 118-27 (2007).
16. Jaddoe, V.W. *et al.* The Generation R Study: design and cohort update 2012. *Eur J Epidemiol* **27**, 739-56 (2012).
17. Kruithof, C.J. *et al.* The Generation R Study: Biobank update 2015. *Eur J Epidemiol* **29**, 911-27 (2014).
18. Bergen, N.E. *et al.* Homocysteine and folate concentrations in early pregnancy and the risk of adverse pregnancy outcomes: the Generation R Study. *Bjog* **119**, 739-51 (2012).
19. Eyles, D. *et al.* A sensitive LC/MS/MS assay of 25OH vitamin D3 and 25OH vitamin D2 in dried blood spots. *Clin Chim Acta* **403**, 145-51 (2009).
20. Gazibara, T. *et al.* Associations of maternal and fetal 25-hydroxyvitamin D levels with childhood lung function and asthma.The Generation R Study. *Clin Exp Allergy* (2015).
21. Touleimat, N. & Tost, J. Complete pipeline for Infinium((R)) Human Methylation 450K BeadChip data processing using subset quantile normalization for accurate DNA methylation estimation. *Epigenomics* **4**, 325-41 (2012).
22. Pidsley, R. *et al.* A data-driven approach to preprocessing Illumina 450K methylation array data. *BMC Genomics* **14**, 293 (2013).
